# Pathogenicity island excision during an infection by *Salmonella enterica* serovar Enteritidis is required for crossing the intestinal epithelial barrier in mice to cause systemic infection

**DOI:** 10.1371/journal.ppat.1008152

**Published:** 2019-12-04

**Authors:** Catalina Pardo-Roa, Geraldyne A. Salazar, Loreani P. Noguera, Francisco J. Salazar-Echegarai, Omar P. Vallejos, Isidora D. Suazo, Bárbara M. Schultz, Irenice Coronado-Arrázola, Alexis M. Kalergis, Susan M. Bueno

**Affiliations:** 1 Millennium Institute on Immunology and Immunotherapy, Departamento de Genética Molecular y Microbiología, Facultad de Ciencias Biológicas, Pontificia Universidad Católica de Chile, Santiago, Chile; 2 Departamento de Endocrinología, Facultad de Medicina, Pontificia Universidad Católica de Chile, Santiago, Chile; University of California Davis School of Medicine, UNITED STATES

## Abstract

Pathogenicity island excision is a phenomenon that occurs in several *Salmonella enterica* serovars and other members of the family *Enterobacteriaceae*. ROD21 is an excisable pathogenicity island found in the chromosome of *S*. Enteritidis, *S*. Dublin and *S*. Typhi among others, which contain several genes encoding virulence-associated proteins. Excision of ROD21 may play a role in the ability of *S*. Enteritidis to cause a systemic infection in mice. Our previous studies have shown that *Salmonella* strains unable to excise ROD21 display a reduced ability to colonize the liver and spleen. In this work, we determined the kinetics of ROD21 excision *in vivo* in C57BL/6 mice and its effect on virulence. We quantified bacterial burden and excision frequency in different portions of the digestive tract and internal organs throughout the infection. We observed that the frequency of ROD21 excision was significantly increased in the bacterial population colonizing mesenteric lymph nodes at early stages of the infective cycle, before 48 hours post-infection. In contrast, excision frequency remained very low in the liver and spleen at these stages. Interestingly, excision increased drastically after 48 h post infection, when intestinal re-infection and mortality begun. Moreover, we observed that the inability to excise ROD21 had a negative effect on *S*. Enteritidis capacity to translocate from the intestine to deeper organs, which correlates with an abnormal transcription of *invA* in the *S*. Enteritidis strain unable to excise ROD21. These results suggest that excision of ROD21 is a genetic mechanism required by *S*. Enteritidis to produce a successful invasion of the intestinal epithelium, a step required to generate systemic infection in mice.

## Author summary

*Salmonella* is a bacterial genus that causes foodborne illnesses worldwide. The ability of *Salmonella* to cause disease is related to the presence of Pathogenicity Islands (PAIs), which are clusters of genes within the bacterial chromosome that are involved in virulence. Interestingly, some PAIs excise and re-integrate into the bacterial chromosome, which is a process probably involved in the capacity of *Salmonella* to cause infection in their hosts. Here we show that the excision of Region of Difference 21 (ROD21), one of the excisable PAIs within the genome of *Salmonella enterica* serovar Enteritidis, occurs with high frequency in the mesenteric lymph node at early stages of infection, suggesting that excision is required by *S*. Enteritidis to reach this organ from the intestinal tract. Accordingly, *S*. Enteritidis strains unable to excise ROD21 are unable to invade intestinal host cells, delaying the infective cycle and showing attenuated virulence. We propose that ROD21 excision *in vivo* is required by *S*. Enteritidis to cross the intestinal barrier, a fundamental step to further colonize deep organs, due to modulation of virulence genes transcription. Thus, ROD21 excision may play an important role in the capacity of the bacteria to cause a successful systemic infection in the mouse. Our data suggest that the excision of PAIs is a mechanism used by *Salmonella* and probably other Gram-negative enterobacteria to modulate the expression of virulence genes and may provide insights to design novel therapies to control the infection caused by these pathogens.

## Introduction

*Salmonella* is a genus of Gram-negative, facultative intracellular enteric pathogens. *Salmonella* enterica subs. enterica (*Salmonella enterica*) is composed by more than 1,531 serotypes, among which *S*. Typhimurium and *S*. Enteritidis are pathogens responsible for most human infections [1]. One of the main features of *Salmonella enterica* is its ability to cause disease in different hosts such as humans, pigs, cattle, birds and mice [2]. In humans, *Salmonella enterica* serovar Enteritidis (*S*. Enteritidis) causes several foodborne diseases, such as gastroenteritis and systemic/persistent disease [3–5]. Although streptomycin pretreatment in the mouse has been used as a surrogate enterocolitis model [6, 7], the natural infection of *S*. Enteritidis in these animals cause systemic and persistent infection, which seems to be a mechanism of bacterial transmission to other hosts, including poultry [8–10]. Therefore, *S*. Enteritidis is considered a relevant zoonotic pathogen that is able to survive inside eukaryotic cells, allowing the development of systemic disease and causing a persistent infection in the host [11–14].

The virulence of these bacterial strains relies on pathogenicity islands (PAIs), which are large clusters of genes (10 to 100 kb) located in the genome of pathogenic bacteria and absent in the genome of non-pathogenic strains of the same or closely related species [15, 16]. Some PAIs have the ability to excise and re-integrate from the bacterial chromosome. This ability is due to the presence of Direct Repeated Sequences (DRS) at their ends [24]. DRS sites are recognized by integrases and excisionases/recombination directionality factors that catalyze the excision, generating an episomal element that contains one copy of the DRS sites (*att*P), while the other copy remains in the chromosome (*att*B) [24]. The spontaneous excision of PAIs has been described in several pathogenic bacteria, such as *E*. *coli* 503, *Legionella pneumophila*, *Yersinia pseudotuberculosis*, *Vibrio cholera* and various *Salmonella enterica* serovars [17–24]. One of the excisable PAI of *Salmonella enterica* is the Region of Difference 21 (ROD21) [22], which was initially found in the chromosome of *S*. Enteritidis, *S*. Gallinarum, *S*. Dublin and *S*. Typhi [25, 26], but more recently it has been found in other serovars such as *S*. Pullorum, *S*. Nitra and *S*. Antatum [27]. ROD21-like islands has also been found in other members of the *Enterobacteriaceae* family that include *Escherichia coli* ETEC-2265, *Klebsiella pneumoniae* 30684, *Klebsiella oxytoca* AR0147 and *Pectobacterium atrosepticum* SCRI1043 [27].

Previous studies have shown that environmental factors can influence the frequency of excision of ROD21. For instance, excision of this PAI increased when *S*. Enteritidis was infecting dendritic cells and macrophages [22]. Also, environmental conditions that mimic the lysosomal core have shown to increase the frequency of excision of ROD21 [28]. Lower pH also increased the excision of ROD21 [28], suggesting that during the infective cycle the exposure of *Salmonella* to reactive oxygen species or pH changes can modulate the excision of this PAI.

ROD21 harbors several genes encoding virulence proteins, such as *SEN1975* that encodes for TlpA, a protein associated with the bacterial ability to cause lethal disease in mice [29]. This PAI also harbors the CDS *SEN1978*, which encodes a putative type IV pilin, and the CDS *SEN1979* (*TraD*) and *SEN1980 (MobA/MobL*), which encodes conjugation system-related proteins [29]. Importantly, ROD21 also harbors *SEN1993*, a gene that codes for a protein similar to H-NS [22]. This latter protein is involved in DNA organization and contributes to the replication, segregation, repair and expression of the bacterial chromosome [30–32]. Previous *in vitro* studies have shown that strains unable to excise ROD21 displayed altered expression of the genes located in this PAI [27, 33], suggesting that excision might also regulate gene transcription. Even though the relationship between PAIs excision and virulence has not been completely elucidated, previous reports suggest that strains of *S*. Enteritidis that are unable to excise ROD21 have a reduced capacity to colonize the liver and spleen of mice, as compared to the WT strain [33]. Also, recent reports performed global genome analyses of *S*. Enteritidis strains showed that an Easter African strain lacking this PAI showed significantly reduced virulence as compared to strains harboring ROD21 [34]. Importantly, the same study showed that strains of *S*. Enteritidis lacking ROD21 displayed an impaired ability to colonize livers and spleens in poultry [34].

To expand on the contribution of the excision of ROD21 to *S*. Enteritidis virulence in mice, we evaluated the *in vivo* occurrence of ROD21 excision during the different stages of the infection caused by *S*. Enteritidis in C57BL/6 mice. We observed that excision of ROD21 occurred at different stages of the infection cycle in mice. However, we observed that this process was significantly increased during the initial colonization of deep organs, such as mesenteric lymph nodes, spleen and liver. Further, we generated a *S*. Enteritidis strain unable to excise ROD21 due to the lack of the genes encoding for the integrase and Recombination Directionality Factor (RDF). We observed that this mutant strain colonizes the gastrointestinal tract as the WT strain, but it showed a major defect in the ability to invade epithelial host cells and survive intracellularly, which results in a delayed capacity to colonize internal organs, such as spleen and liver. These results suggest that the excision of ROD21 is required to promote the invasion of host cells by *Salmonella*, which is required for the subsequent bacterial dissemination from the digestive tract to colonize internal organs.

## Results

### ROD21 excision is significantly increased during infection of internal organs

**Fig 1:**
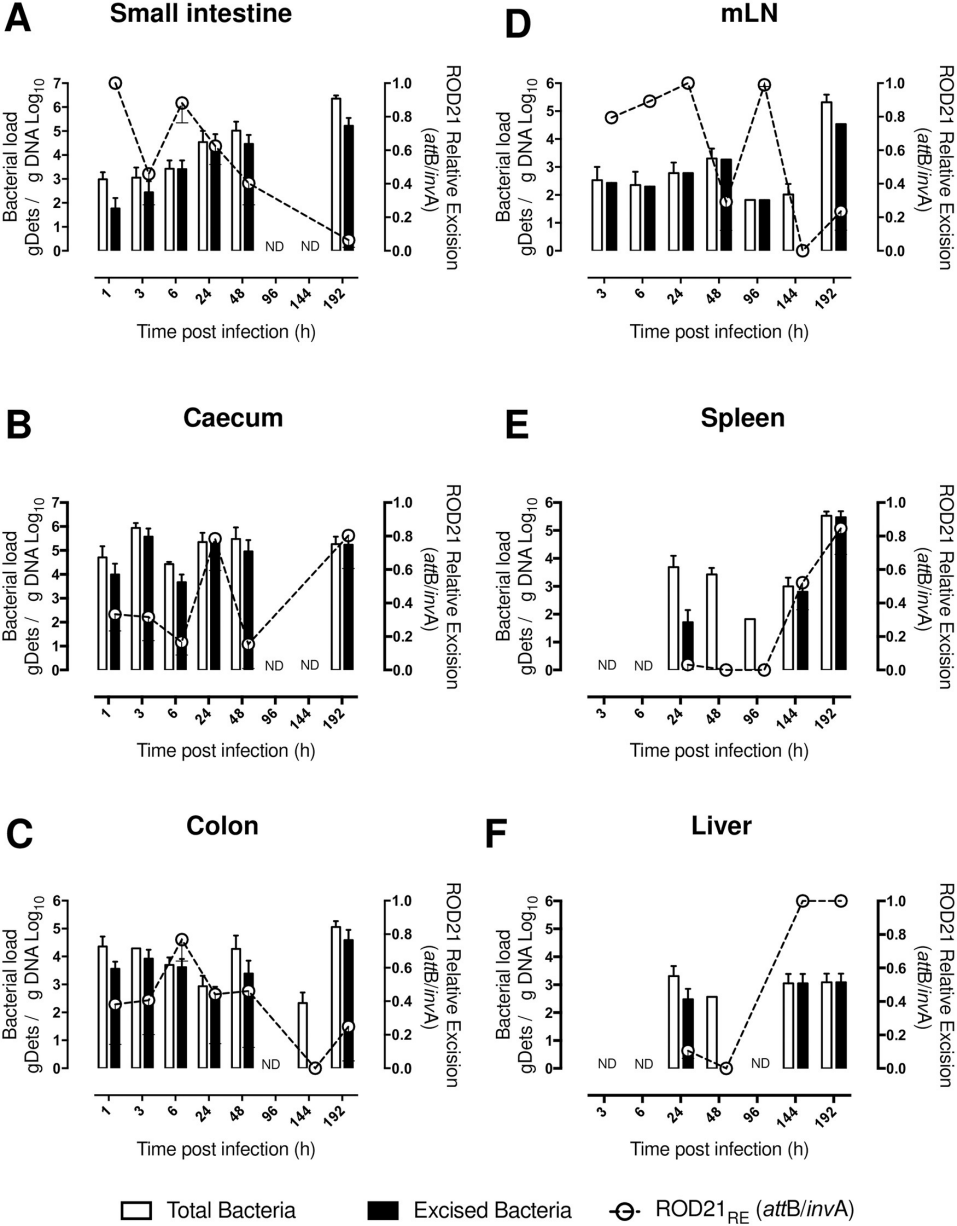
Determination of ROD21 excision from *S*. Enteritidis chromosome during the infective cycle. After intragastric (i.g.) infection of C57BL/6 mice, quantification of total *Salmonella* gDets and absolute ROD21 excision in different portions of the gastrointestinal tract and deep organs was performed by the quantification of the *invA* (total bacteria, empty bars) or *attB* (excised bacteria, black bars) sequences, by qPCR. gDNA from small intestine (A), caecum (B), colon (C), mLN (D), spleen (E) and liver (F) were purified and the presence of *S*. Enteritidis-WT chromosomes was identified, number of copies for each sequence were normalized per μg of gDNA. Empty circles and dotted line over each time post-infection are the relative ROD21 excision value (_RE_ROD21 = attB gDets/ invA gDets). The assay included 4 mice for each time. 2-way ANOVA with Tukey´s post-test α = 0.05; *p<0.05, **p<0.005, ***p<0.0005, ****p<0.0001.

To establish the frequency of excision of ROD21 during the infective cycle, groups of C57BL/6 mice were intragastrically (i.g.) infected with 1x10^6^ CFUs of *S*. Enteritidis-WT and different portions of the digestive tract, internal organs and feces were recovered at various time points after infection (1, 3, 6, 24, 48, 96, 144, and 192 h post-infection, hpi). Total genomic DNA (gDNA) was purified and *invA* sequence was detected by qPCR to quantify the total number of bacterial chromosomes in each organ. The *att*B sequence was also determined by qPCR to quantify the number of bacterial chromosomes, or genomic determinants (gDets), that underwent ROD21 excision (see Materials and methods). Correlation assay and Spike and recovery tests showed that this molecular approach to quantify *S*. Enteritidis-WT gDets in tissues significantly correlates with the amount of colony forming units (CFU) recovered from the same infected tissues (S1 Fig and S1 Table). As shown in Fig 1A–1C, *S*. Enteritidis-WT gDets that underwent ROD21 excision were detected in different portions of the gastrointestinal tract, between 1 to 48 hpi. It is noteworthy that the relative excision of ROD21 (_RE_RO21 = *att*B/*invA*) calculated in these tissues varied from 0.3 to 0.7 (Fig 1). After 96 hpi, the number of total *S*. Enteritidis-WT gDets detected was significantly reduced in the digestive tract, however, it was detected again at 192 hpi. Importantly, at this latter time post-infection we observed values of _RE_ROD21 equivalent to those observed at early times after infection (1–6 hpi, Fig 1). These results suggest that ROD21 excision from the chromosome occurs evenly along the digestive tract. ROD21 excision was also evaluated in the gastric content and feces (S2 Fig). On feces, we observed a high number of total *S*. Enteritidis-WT gDets at all the time post infection measured, consistent with a variable _RE_ROD21 (between 0.23–1.0, S2 Fig).

In mLN, total *S*. Enteritidis-WT gDets could be detected from 3 to 144 hpi (Fig 1D). However, between 96 and 144 hpi a significant reduction in the number of *S*. Enteritidis-WT gDets was observed. At 192 hpi the number of total *S*. Enteritidis-WT gDets significantly increased, suggesting re-infection of the mLN. Importantly, from 3 to 96 hpi, a high number of *S*. Enteritidis-WT gDets that underwent excision were detected (_RE_ROD21 between 0.53 to 1, Fig 1D). However, the level of ROD21 excision at 192 hpi, when re-infection occurs, was low (_RE_ROD21 0.2–0.35, Fig 1). This observation suggests that in the second round of mLN infection the excision of ROD21 was not promoted, as observed at earlier times after infection.

In spleen and liver, the presence of *S*. Enteritidis started to be detected from 24 to 48 hpi (Fig 1E and 1F). At these times post infection, only a minor fraction of *S*. Enteritidis-WT gDets that underwent excision were detected. In agreement, the _RE_ROD21 calculated in these organs at these times post infection was also low. Then, a reduction in the number of total *S*. Enteritidis-WT gDets was observed at 96 hpi in both organs, but a significant increase of *S*. Enteritidis-WT gDets was detected at 144 and 192 hpi. Interestingly, at these time points after infection, a significant fraction of *S*. Enteritidis-WT gDets underwent excision (_RE_ROD21 between 0.34 to 1, Fig 1E and 1F). Additionally, in the gallbladder, total *S*. Enteritidis-WT genomes also started to be detected from 24 hpi until 96 hpi. Importantly, only at 48 hpi a high number of *S*. Enteritidis-WT genomes that underwent ROD21 excision was detected (_RE_ROD21 between 0.73 to 0.84, S2 Fig). These results suggest that excision of ROD21 was performed with high frequency at specific time points after infection in deep organs: in mLN occurs at initial stages of infection, while in spleen and liver occurs at late stages of the infective cycle. According to these results, it is possible that the excision of ROD21 might play an important role during the processes required to promote infection of these organs, especially at times where _RE_ROD21 is high.

### ROD21 excision is required by *S*. Enteritidis to reach internal organs from the gastrointestinal tract

To support the notion that the excision of ROD21 contributes to the capacity of *S*. Enteritidis to infect the digestive tract and deep organs at specific stages of the infective cycle, we generated a *S*. Enteritidis strain lacking both the integrase and the RDF coding genes. As a result, these strains are unable to excise ROD21 (S3C Fig). Importantly, there were no growth differences between *S*. Enteritidis-WT and *S*. Enteritidis-Δ*int*Δ*rdf*, neither in standard nor in minimal culture media (S3A and S3B Fig). We did not observed differences either in the growth of both the WT or Δ*int*Δ*rdf* at 43°C (S3A and S3B Fig).

Next, groups of C57BL/6 mice were i.g. infected either with *S*. Enteritidis-WT, *S*. Enteritidis-Δ*int*Δ*rdf* strains or treated with PBS. We observed that 100% of mice infected with *S*. Enteritidis-WT strain died before 10 days, while mice infected with *S*. Enteritidis-Δ*int*Δ*rdf* strain started to die only after 11 days post-infection. Importantly, 50% of mice infected with *S*. Enteritidis-Δ*int*Δ*rdf* strain lived, confirming that impaired ROD21 excision affects the virulence of *S*. Enteritidis (S3D Fig). Weight was registered on a daily basis and, as expected, mice infected with the *S*. Enteritidis-WT strain showed significant weight loss as compared to mice infected with *S*. Enteritidis-Δ*int*Δ*rdf* or the control uninfected group (S3 Fig).

**Fig 2:**
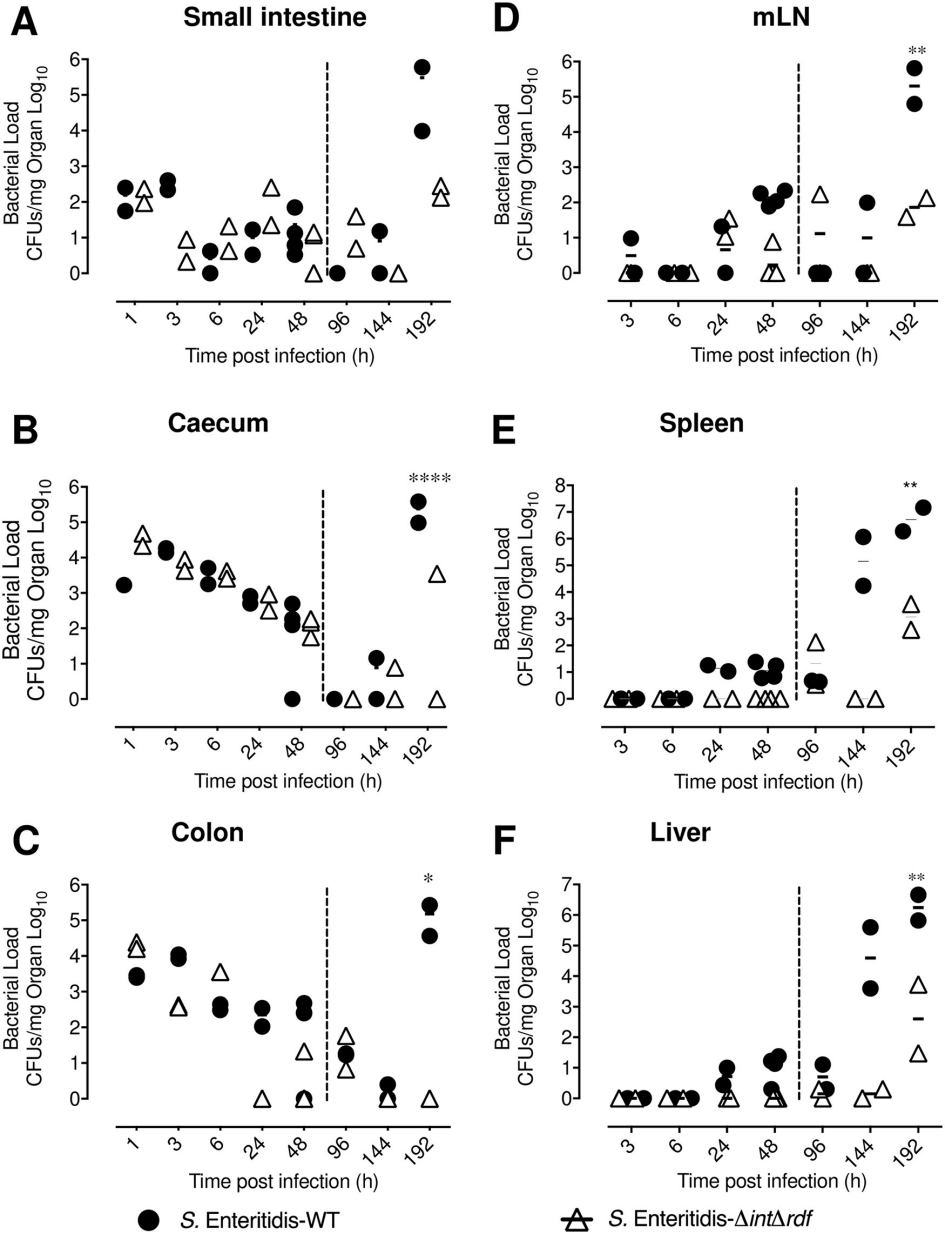
*S*. Enteritidis ability to perform systemic infection requires excision of ROD21. C57BL/6 mice were i.g. infected with 1x10^6^ CFU of *S*. Enteritidis-WT (black circle) or *S*. Enteritidis-Δ*int*Δ*rdf* (empty triangle). At different times post infection, the bacterial load was evaluated on small intestine (A), caecum (B), colon (C), mLN (D), spleen (E) and liver (F). The assay included two mice at all times post-infection, except at 48 hpi, which included four mice. Comparisons of bacterial loads between different times post-infection were analyzed by 2-way ANOVA with Tukey´s post-test. *p<0.05, **p<0.005, ***p<0.0005, ****p<0.0001. α = 0.05 between Δ*int*Δ*rdf* and WT at 192 hpi.

To determine in which part of the infective cycle the excision of ROD21 is required for *S*. Enteritidis infective cycle, bacterial burdens were quantified at 1, 3, 6, 24, 48, 96, 144, and 192 hpi in the gastrointestinal tract and in deep organs of mice infected either with *S*. Enteritidis-WT or *S*. Enteritidis-Δ*int*Δ*rdf*. As shown in Fig 2A, the bacterial passage across the small intestine occurred rapidly for both strains, remaining in low or undetectable levels after 48 hpi. Caecum and colon were also colonized at early times post infection (Fig 2B and 2C). Initially, high bacterial loads for both strains were observed in the caecum, with a significant reduction at 96 hpi. In the colon, *S*. Enteritidis-Δ*int*Δ*rdf* strain was undetected at 24 hpi but showed a discrete increase at 96 hpi. Also, in these tissues a significant increase for the amount of *S*. Enteritidis-WT was observed at 192 hpi, probably due to the second round of infection in the gastrointestinal tract caused by the bacterial excretion from the gallbladder. However, *S*. Enteritidis-Δ*int*Δ*rdf* was unable to generate this re-infection process, suggesting that a deficiency to excising ROD21 affects the ability of *S*. Enteritidis to cause a systemic dissemination in the host.

An equivalent quantification of the bacterial load was performed in deep organs. As shown in Fig 2D, 2E and 2F, the amount of *S*. Enteritidis-Δ*int*Δ*rdf* was significantly reduced in mLN, spleen and liver at late stages of the infection (144 and 192 hpi), as compared to *S*. Enteritidis-WT. However, the bacterial load of *S*. Enteritidis-Δ*int*Δ*rdf* started to increase at 192 hpi, suggesting that lack of ROD21 excision could significantly delay the progression of the infective cycle of *S*. Enteritidis.

### ROD21 excision is required by *S*. Enteritidis to cross the intestinal epithelial barrier

**Fig 3:**
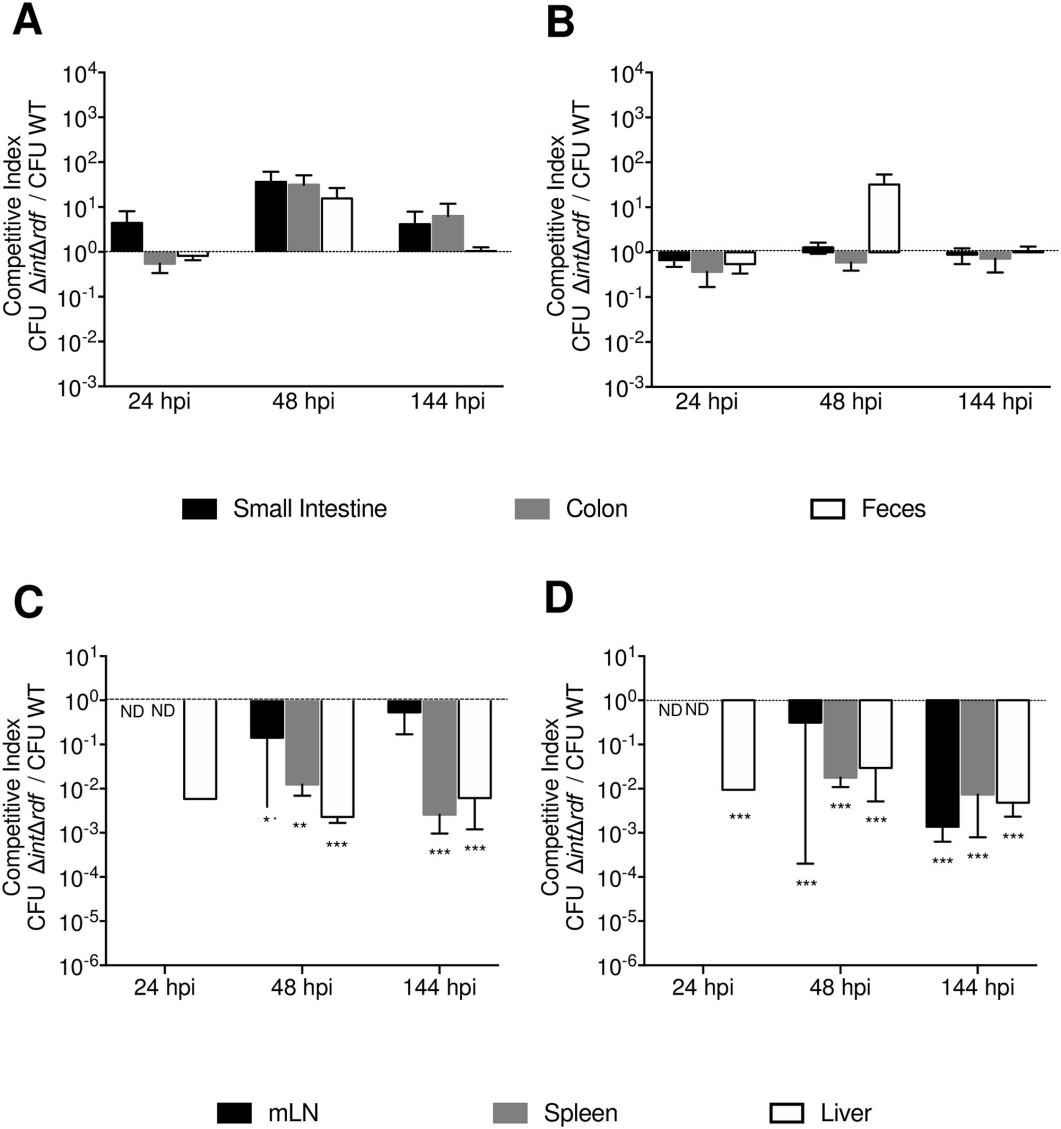
ROD21 excision is required to cross intestinal epithelial barrier and initiate colonization of internal organs. *In vivo* competition assay for *S*. Enteritidis-Δ*int*Δ*rdf* versus *S*. Enteritidis-WT were performed using a total of 10^6^ CFUs of each strain administered at a 1:1 ratio i.g. Mice were euthanized and organs recovered at 24, 48 and 144 hpi. CI values were calculated as the mean ratio of *S*. Enteritidis-Δ*int*Δ*rdf* to *S*. Enteritidis-WT CFUs, normalized to the input ratio and converted logarithmically (output ratio). Competitions assays were performed in intestinal tissues (A and B) and deep organs (C-D). Black line indicates the input ratio. A and C graphs show the results obtained for infection of *S*. Enteritidis-Δ*int*Δ*rdf*::*aph* / *S*. Enteritidis-WT::*cat*. B and D graphs show the results obtained for infection of *S*. Enteritidis-Δ*int*Δ*rdf*::*cat* / *S*. Enteritidis-WT::*aph*. The assay included 6 mice at all times post-infection, statistical significance was determined by using a 2-tailed Student’s *t* test, and asterisks indicate that normalized output ratios were significantly different from the equivalent ratio in the inoculum (*p<0.05, **p<0.005, ***p<0.0005, ****p<0.0001).

Competition assays were performed to evaluate what stage of the infective cycle requires the excision of ROD21 to support the progression of the infection in mice. Using a *S*. Enteritidis strains with either kanamycin or chloramphenicol resistance genes, mice were i.g infected with a mixture of WT and Δ*int*Δ*rdf S*. Enteritidis (1:1 ratio). After 24 hpi the amount of each bacteria strain was quantified in the small intestine, caecum, colon, feces, mLN, blood, liver and spleen. To compare the ability of these strains to colonize tissues, the competition index (CI) was determined as described in Materials and Methods. As shown in Fig 3A, for all portions of the gastrointestinal tract, the CI for the Δ*int*Δ*rdf* v/s WT was close to 1 at the various time points evaluated. In contrast, CI values obtained in internal tissues at 48 and 144 hpi were below 1 (Fig 3B), indicating that a higher amount of the WT strain than the Δ*int*Δ*rdf* was recovered from these tissues. Importantly, equivalent results were obtained independently of the antibiotic resistance genes incorporated into either WT or Δ*int*Δ*rdf* strains (Fig 3).

Invasion of host cells by *S*. Enteritidis is a key step for crossing the epithelial barrier and reaching the sub-epithelial dome. We evaluated whether the excision of ROD21 was required by *S*. Enteritidis to invade the intestinal tissue. Tissues from mice infected with both *S*. Enteritidis-WT and *S*. Enteritidis-Δ*int*Δ*rdf* (1:1 ratio) were recovered, disaggregated and treated with gentamicin for 1 h to kill extracellular bacteria. Then, intracellular bacteria were released and grown in media supplemented with kanamycin or chloramphenicol. Notably, while *S*. Enteritidis-WT was obtained from these infected tissues, *S*. Enteritidis-Δ*int*Δ*rdf* failed to be recovered from any of the tissues evaluated (S4 Fig). Therefore, it was not possible to calculate a CI for intracellular bacteria in the tissues and times included in these assays. Importantly, equivalent results were obtained regardless of the antibiotic-resistant gene incorporated into the WT or Δ*int*Δ*rdf* strains. These results suggest that excision of ROD21 is required by *S*. Enteritidis to cross the epithelial barrier in the gastrointestinal tract to generate systemic infection in mice.

### Excision of ROD21 is required by *S*. Enteritidis to invade intestinal epithelial cells, to colonize internal organs and to sustain systemic dissemination

To test the contribution of ROD21 excision to the ability of *S*. Enterititis to survive intracellularly in infected mice, *in vivo* assays were performed using *S*. Enteritidis-WT and two different strains unable to excise ROD21: *S*. Enteritidis-Δ*int*Δ*rdf* and *S*. Enteritidis-ΔDRSΔ*int* [33]. Furthermore, a *S*. Enteritidis strain lacking the whole PAI (*S*. Enteritidis-ΔROD21) and the complemented strain (*S*. Enteritidis-ΔROD21::ROD21) [28] were also included as controls. Mice were i.g. infected with each strain and at 48 hpi and 144 hpi single cell suspensions were obtained from the small intestine, colon, mLN, blood, liver and spleen. Then, total bacteria were determined by direct seeding of detergent-treated cells in LB plates, while intracellular bacteria were determined by a gentamicin protection assay performed in cells obtained from the recovered organs. As described above, the transit of all the bacterial strains across the intestinal tract was equivalent at early stages of infection (S5A–S5C Fig). However, at 144 hpi we observed reduced loads of *S*. Enteritidis-ΔROD21, *S*. Enteritidis-ΔDRSΔ*int* and *S*. Enteritidis-Δ*int*Δ*rdf* in intestinal tissues, as compared to *S*. Enteritidis-WT and *S*. Enteritidis-ΔROD21::ROD21 (S5A–S5C Fig). In feces, at 48 hpi we observed reduced burden of *S*. Enteritidis-ΔDRSΔ*int* and *S*. Enteritidis-Δ*int*Δ*rdf*, but at 144 hpi the amounts of these mutant strains in feces were equivalent to *S*. Enteritidis-WT, *S*. Enteritidis-ΔROD21 and *S*. Enteritidis-ΔROD21::ROD21.

In mLN, blood, spleen, liver and gallbladder low loads of total *S*. Enteritidis-WT were detected at 48 hpi, with a significant increase at 144 hpi. However, no load of *S*. Enteritidis-ΔDRSΔ*int* or *S*. Enteritidis-ΔROD21 were detected at 48 hpi (S5E–S5I Fig). Further, significantly fewer loads of these mutant strains were detected at 144 hpi in the spleen, liver and gallbladder, as compared to the WT strain. Importantly, complementation of ROD21 in *S*. Enteritidis-ΔROD21 restored the WT phenotype (S5 Fig).

**Fig 4:**
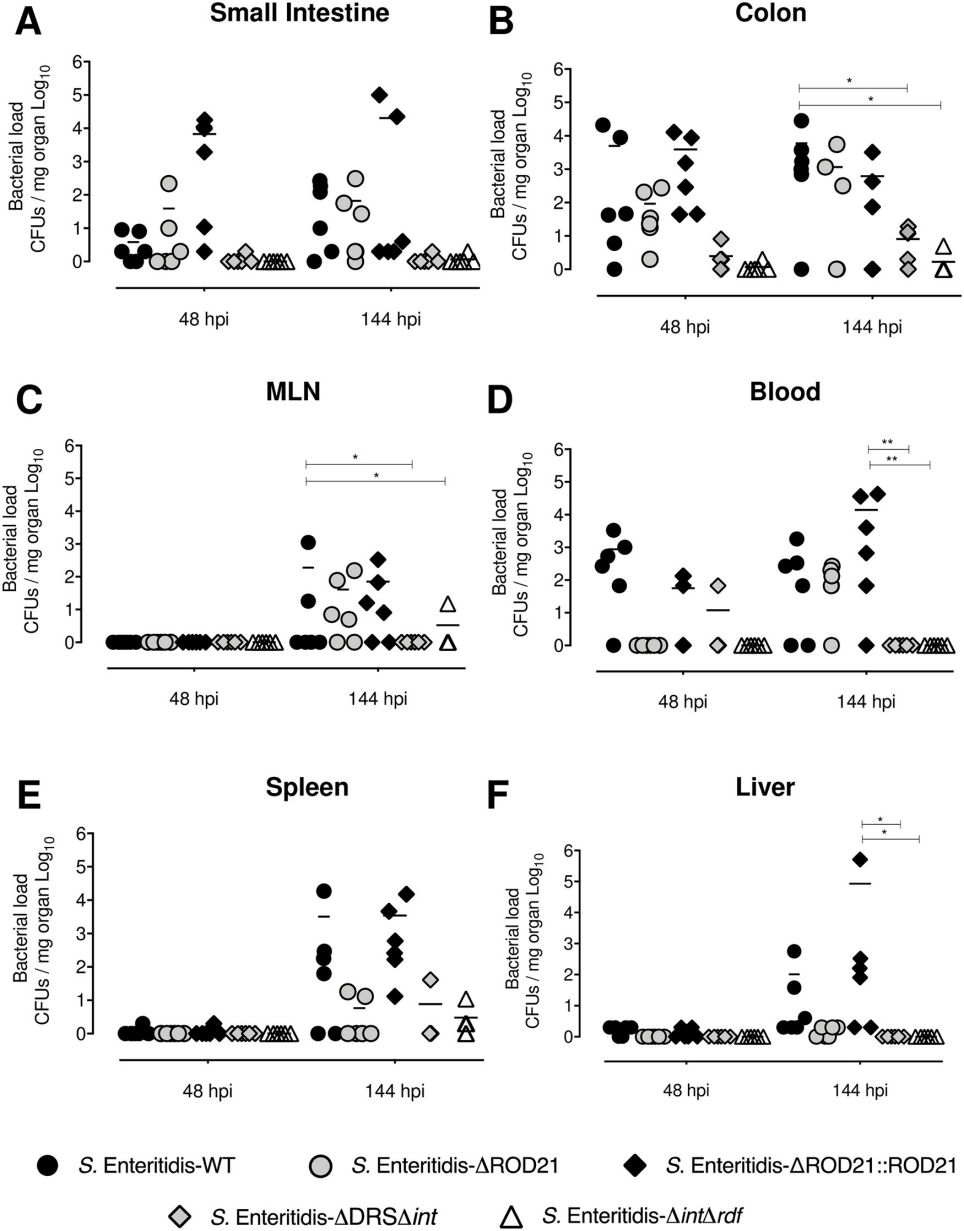
ROD21 excision is required for the intracellular colonization of *S*. Enteritidis in the intestinal epithelium during the infective cycle. C57BL/6 mice were i.g. infected with 1x10^6^ CFU of *S*. Enteritidis-WT (black circles), *S*. Enteritidis-ΔROD21 (grey circles), *S*. Enteritidis-ΔROD21::ROD21 (black diamonds), *S*. Enteritidis-ΔDRSΔ*int* (grey diamonds) or *S*. Enteritidis-Δ*int*Δ*rdf* (empty triangles). At 48 and 144 hpi a gentamicin protection assay was performed and intracellular loads were evaluated on small intestine (A), colon (B), mLN (C), blood (D), spleen (E) and liver (F). The assay included 6 mice at all times post infection. Comparisons of bacterial loads between different times post-infection were analyzed by 2-way ANOVA with Tukey´s post-test α = 0.05. *p<0.05, **p<0.005, ***p<0.0005, ****p<0.0001.

Regarding the ability of bacteria to invade intracellularly the intestinal epithelium, no differences were found between the WT, ΔROD21 and ΔROD21::ROD21 strains (Fig 4A and 4B). However, *S*. Enteritidis-Δ*int*Δ*rdf* and *S*. Enteritidis-ΔDRSΔ*int* showed a severe impairment to invade the intestinal epithelium at 48 and 144 hpi, as compared to the other strains (Fig 4A and 4B). In mLN, significant differences were observed in the ability of *S*. Enteritidis-Δ*int*Δ*rdf* and *S*. Enteritidis-ΔDRSΔ*int* to intracellularly colonize this tissue at 144 hpi, as compared to WT, ΔROD21 and ΔROD21::ROD21 strains (Fig 4C). In blood, we observe no presence of the *S*. Enteritidis-Δ*int*Δ*rdf* strain at 144 hpi. In spleen and liver, we observe a defect if the ability of ΔROD21, ΔDRSΔ*int* and -Δ*int*Δ*rdf* strains to intracellularly colonize these organs at 144 hpi (Fig 4E and 4F). In summary, these data suggest that genes in ROD21 might be required to cause a successful invasion of tissues during systemic infection, but the excision of ROD21 may play additionally a key role during *S*. Enteritidis intracellular invasion of intestinal cells, promoting the translocation of the bacteria from the intestine to facilitate systemic dissemination.

To determine whether the excision process is required just during the translocation of bacteria from the intestine to internal organs, or it is required also during invasion of internal organs, we performed intraperitoneal infections. When survival was determined after infection with the different strains of *S*. Enteritidis, significantly increased survival was observed for mice challenged with mutant strains unable to excise ROD21 as compared to those infected with the WT strain. However, eventually, all infected mice died at early times post infection (S6 Fig). When comparing these results with the survival rates observed after an oral infection (S3 Fig), our results suggest that ROD21 excision contributes more significantly to the ability of *S*. Enteritidis to translocate from the intestinal tract to deeper organs than to the capacity to disseminate and cause systemic infection.

### Transfer of ROD21 improves the ability of *S*. Typhimurium to cause infection in mice

**Fig 5:**
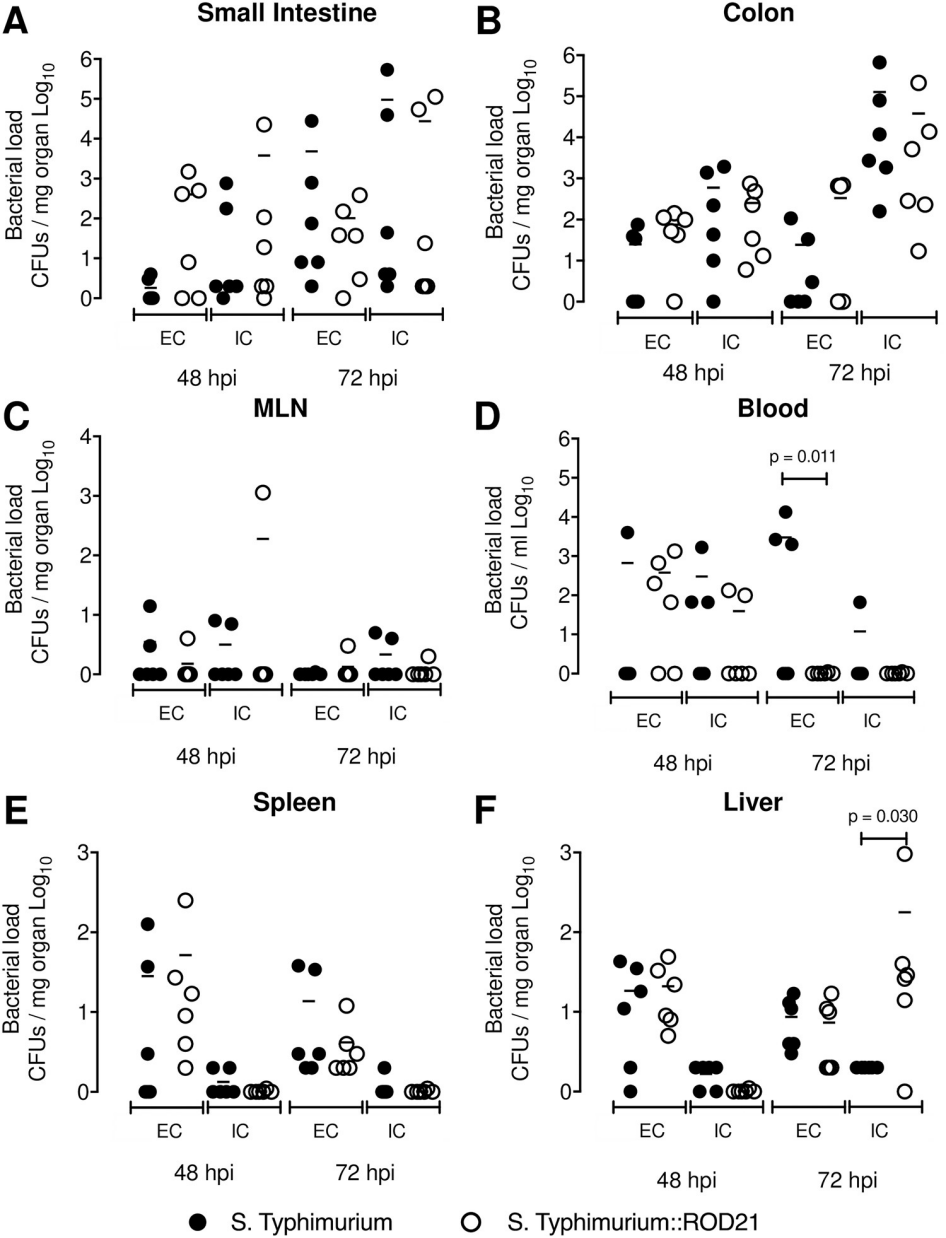
ROD21 improves the intracellular survival of *S*. Typhimurium on liver. C57BL/6 mice were i.g. infected with 1x10^5^ CFU of *S*. Typhimurium-WT (black circles) or *S*. Typhimurium::ROD21 (empty circles). At 48 and 72 hpi extra and intracellular loads were evaluated on small intestine (A), colon (B), mLN (C), blood (D), spleen (E) and liver (F). The assay included 6 mice at all times post infection. Comparisons of bacterial loads between different times post-infection were analyzed by 2-way ANOVA with Tukey´s post-test α = 0.05.

We also evaluated whether the transfer of ROD21 into another *Salmonella* serovar that naturally lacks this PAI can modulate its capacity to cross the epithelial barrier and infect internal organs. As shown previously by Salazar-Echegarai et al., a *S*. Typhimurium strain that received a copy of ROD21 by conjugation (*S*. Typhimurium::ROD21) displayed an increased capacity to cause systemic disease [28]. Mice were i.g. infected with 1x10^5^ CFUs of each strain and after 48 hpi and 72 hpi a gentamicin protection assay was performed to quantify the extracellular and intracellular bacteria in the organs described above. No differences were found on intestinal passage or systemic dissemination between both strains (Fig 5 and S7 Fig). However, we found differences at 72 hpi between extracellular bacteria on blood of mice infected with *S*. Typhimurium-WT, as compared to mice infected to *S*. Typhimurium::ROD21, and in the intracellular load of *S*. Typhimurium::ROD21 in the liver, as compared to loads found in mice infected with the WT strain (Fig 5). These results suggest that the presence of ROD21 enhances the ability of *Salmonella* to reach deep organs not only in *S*. Enteritidis, but in other serovars as well.

### ROD21 excision modulates the expression of virulence genes in *S*. Enteritidis

**Fig 6:**
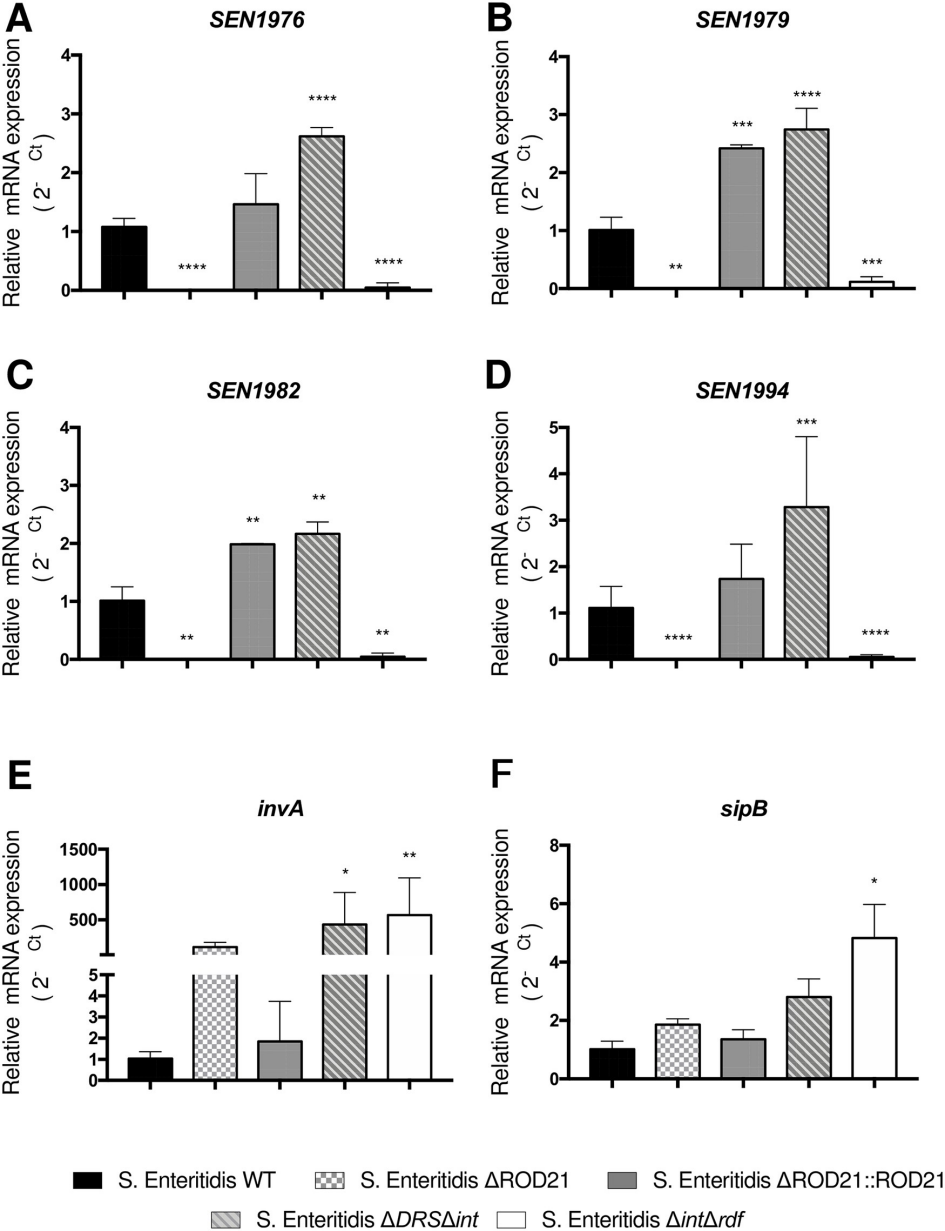
ROD21 excision affects the transcription of genes within *Salmonella* pathogenicity islands. Transcription of genes within ROD21: *SEN1976* (A), *SEN1979* (B), *SEN1982* (C) and *SEN1994* (D) or, within SPI-1: *invA* (E) or *sipB* (F) were evaluated on RNA samples from the inoculums of *S*. Enteritidis-WT, *S*. Enteritidis-ΔROD21, *S*. Enteritidis-ΔROD21::ROD21, *S*. Enteritidis-Δ*DRS*Δ*int* or *S*. Enteritidis-Δ*int*Δ*rdf*. Assay was performed by triplicate. 2-way ANOVA with Tukey´s post-test α = 0.05. *p<0.05, **p<0.005, ***p<0.0005, ****p<0.0001.

Previous studies with *S*. Enteritidis-ΔDRSΔ*int* have suggested that the impairment of ROD21 excision can modulate the expression of genes within ROD21 [27]. Therefore, we evaluated whether *S*. Enteritidis-Δ*int*Δ*rdf* strain also showed an altered gene expression, equivalent to *S*. Enteritidis-ΔDRSΔ*int*, and whether impairment of ROD21 excision affects the transcription of genes within ROD21 and genes in other pathogenicity islands that are required for invasion of epithelial cells and the establishment of a systemic infection in mice. RNA samples were obtained from *S*. Enteritidis-WT, *S*. Enteritidis-Δ*int*Δ*rdf*, *S*. Enteritidis-ΔROD21, *S*. Enteritidis-ΔROD21::ROD21 and *S*. Enteritidis-ΔDRSΔ*int* (all of them grown in LB medium). Then, a RT-qPCR was performed to evaluate expression of ROD21 genes within ROD21 that are known to contribute to the ability of *S*. Enteritidis to cause systemic disease (*SEN1970*, *SEN1975*, *SEN1976*, *SEN1976*, *SEN1980*, *SEN1982*, *SEN1993*, *SEN1994* and *SEN1998)* [35]. Significant differences were observed in the transcription of genes within ROD21 between strains unable to excise ROD21 and between *S*. Enteritidis-WT and *S*. Enteritidis-ΔROD21::ROD21. As shown in Fig 6 and S8 Fig, *S*. Enteritidis-ΔDRSΔ*int* strain had a higher expression of the genes located within ROD21, which is in agreement to our previous report [33]. However, we did not detect transcription of ROD21 genes in *S*. Enteritidis-Δ*int*Δ*rdf* (Fig 6 and S8 Fig). We also examined the transcription of SPI-1 and SPI-2 genes that are related to the ability of *Salmonella* to cross the epithelial barrier during early stages of infective cycle and survive inside *Salmonella*-containing vacuole (SCV). We evaluated the expression of *invA* and *sipB* (located on SPI-1), as well as *spiA* and *spiC* (located on SPI-2) in the different *S*. Enteritidis strains used in this study. We found that *S*. Enteritidis-Δ*int*Δ*rdf*, *S*. Enteritidis-ΔROD21 and *S*. Enteritidis-ΔDRSΔ*int* had a significant increase on *invA* transcription (p<0.0001), as compared to *S*. Enteritidis-WT or *S*. Enteritidis-ΔROD21::ROD21 (Fig 6).

**Fig 7:**
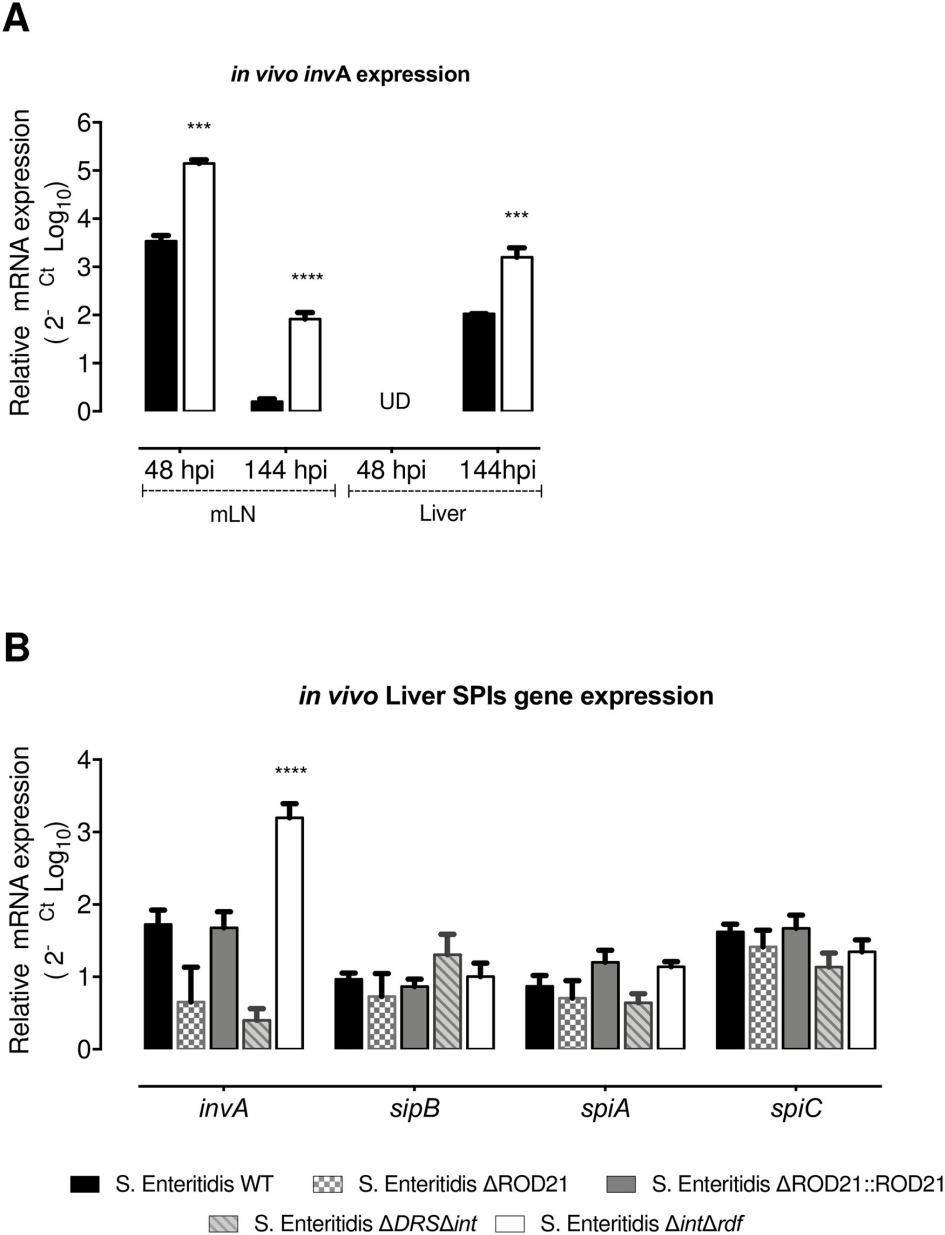
*invA* transcription *in vivo* is regulated by the excision of ROD21. (A) *invA* transcription was quantified on mLN and liver at 48 and 144 hpi for *S*. Enteritidis-WT and *S*. Enteritidis-Δ*int*Δ*rdf*. (B) RNA transcription of i*nvA*, *sipB*, *spiA* and *spiC* were evaluated on samples from livers of infected mice infected with *S*. Enteritidis-WT, *S*. Enteritidis-ΔROD21, *S*. Enteritidis-ΔROD21::ROD21, *S*. Enteritidis-ΔDRSΔ*int* or *S*. Enteritidis-Δ*int*Δ*rdf*. Relative expression was normalized by expression of the infective inoculum. 2-way ANOVA with Tukey´s post-test α = 0.05, n = 4 mice/group. ***p<0.0005, ****p<0.0001.

To evaluate the effect of ROD21 excision on gene transcription when the bacteria is infecting deep organs, we evaluated the transcription of *invA* and ROD21 genes in the mLN and liver from mice infected with *S*. Enteritidis-WT and *S*. Enteritidis-Δ*int*Δ*rdf* at 48 hpi and 144hpi. We found that both strains increased the transcription of *invA* in the mLN at 48 and 144 hpi, as compared to the inoculum grown in LB medium. However, the level of *invA* transcription was significantly higher in *S*. Enteritidis-Δ*int*Δ*rdf* (Fig 7). In the liver, no expression of *invA* was detected at 48hpi in either strains, but it was detected at 144 hpi. Again, *S*. Enteritidis-Δ*int*Δ*rdf* showed a significant increase in *invA* transcription as compared to WT strain. Given that at 144 hpi we were able to detect all mutant strains on liver, and the _RE_ROD21 was near to 1 in this organ, we evaluated the expression of *invA*, *sipB*, *spiA*, *spiC* and ROD21 genes at this time post-infection. We showed that despite similar clinical behavior, mice infected with mutant strains had differences in *invA* transcription, but not in other genes related to SPI-1 and SPI-2 (Fig 7). Unfortunately, it was not possible to evaluate the transcription of ROD21 genes in organs of infected mice using the RT-qPCR approach.

## Discussion

Several members of *Enterobacteriaceae* family have on their chromosome a type of excisable PAIs denominated ROD21-like islands [26, 27]. This is the case of *Salmonella sp* (ROD21 and SPI-7) [23], *Escherichia coli* UPEC (PAI I536, PAI II536, and PAI V536) [36], *Klebsiella pneumoniae* (GIE492) [37], *Vibrio cholera* (VPI-2 VSP-I and VSP-I) [38], and *Pectobacterium atrosepticum* (HAI2) [39]. These islands have significant structural conservation, displaying an unusual G + C profile, are inserted downstream a tRNA gene (usually Asp or Ser), have DRS at the 3´end of the PAI and typically encode paralogs of H-NS (hnsB) and/or an H-NS antagonist [26, 40]. These data suggest that excisable PAIs are a conserved cluster of genes related to the family *Enterobacteriaceae*, and that excision might be an important event related with HGT and/or regulation of gene expression [24, 35]. However, the role of the excision process in virulence has not been elucidated. In this study, we have evaluated whether excision of ROD21 occurs in the infective cycle and whether this process is required by *S*. Enteritidis to generate a successful infection in mice.

ROD21 is a PAI described for the first time in 2008 [26]. Initially, it was found in the chromosome of the reference strain *S*. Enteritidis PT4 and absent on some Easter and Western African isolates [26, 34] that are less virulent than the strain that have this PAI. Importantly, *S*. Enteritidis strains lacking ROD21 have a defect to colonize the liver and spleen, both in mice and poultry [22, 34]. It suggests that the proteins encoded by genes within ROD21 have a role in the virulence of *S*. Enteritidis. Given that our previous results indicate that ROD21 excision affects the capacity *S*. Enteritidis to cause a systemic infection and colonization of the liver and spleen of mice [22, 33], we postulate that the ability of ROD21 to excise from the chromosome plays a role in the virulence of this serovar. In this study, we have deepened the understanding of ROD21 excision during the infection cycle of *S*. Enteritidis in mice.

Our results showed that the *in vivo* excision of ROD21 increases from the beginning of the infection, as compared with the initial inoculum. We also describe here that this is a dynamic process that occurs across the gut, with a stable population able to excise ROD21. However, our results suggest that excision would not be required for early stages of intestinal adaptation. Notably, the main mechanism used by *Salmonella* to adapt to environmental conditions during the initial passage through the host gut are encoded in the core genome, which acts independent of the genes acquired by HGT, including the virulence genes encoded in SPIs. These regulatory systems encoded within these SPIs comprise, among others, the activation of ATR systems [41–43], systems related to survival under limitation of nutrient step [44, 45], tolerance to hyperosmolarity and anaerobic environments [46–48], as well as quorum sensing [49]. Although initially these systems are responsible for the adaptation to the adverse intestinal environment of the host, some of them (such as the two-component systems PhoPQ and PmrAB, or the OmpR protein) are also responsible for the activation of genes encoded within the pathogenicity islands and other HGT elements, to ensure the passage through the intestinal barrier [50–52].

**Fig 8:**
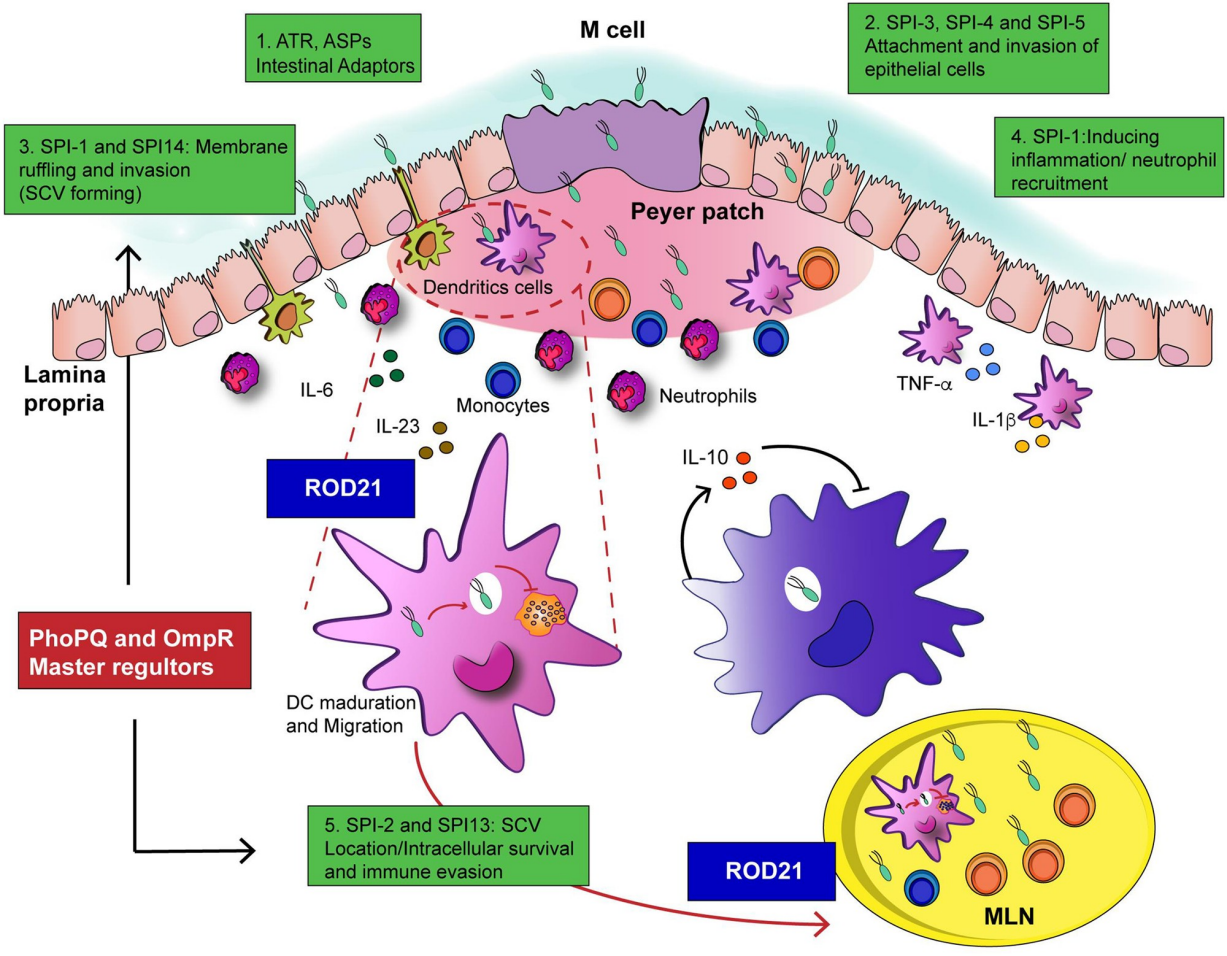
Proposed mechanism of ROD21 interactions during the infective cycle of *S*. Enteritidis in mice. **1.** *Salmonella* is adapted to the stress conditions on the luminal environment by the activation of the ATR, ASP and masters regulators induced by the acidity, osmolarity and temperature; **2.** Then, induces the attachment to intestinal epithelium mediated by the virulence genes encoded on SPI-1 to SPI-5, that also favor the biofilm synthesis. **3.** Internalization in the intestine occurs into the *Sallmonella*-Containing -Vacuole (SVC) on non- phagocytic cells and into the Dendritic Cells (DCs) associated to epithelium. At this point, excision of ROD21, could be important to regulate the expression of genes in SPI-1 in order to favor the survival inside SCV and migration to mLN. **4.** Effectors of SPI-1 induces a proinflammatory environment aiming to induce the phagocytic cells to migrate. **5.** *Salmonella* is able to infect and survive inside phagocytic cells, mainly in DCs and macrophages and to evade the immune system aided by the expression of some SPI-1 effectors and the activation of the T3SS-2 in SPI-2. Here the excision of ROD21 is probably required to regulate the expression of genes inside ROD21 and other SPIs, even the supercoiling of DNA.

We proposed that excision of ROD21 is strongly related to the beginning of the systemic phase of infection. We describe here that *S*. Enteritidis strains unable to excise ROD21 showed a defect in their capacity to reach, colonize and replicate in several deep organs, but had an equivalent ability to colonize the gastrointestinal tract at early times, as compared to WT strain. These observations suggest that the excision process is important to translocate from the intestine to deep organs, to establish a successful systemic infection. In fact, only the *S*. Enteritidis strains unable to excise ROD21, but not *S*. Enteritidis-ΔROD21, showed a severe defect to survive intracellularly in the gastrointestinal tract after oral infection in mice and cause mortality, but intraperitoneal inoculation caused a severe disease that cause higher mortality at earlier times post-infection. These results support the notion that the process of ROD21 excision is required by *S*. Enteritidis to cross the intestinal epithelial barrier. It is possible that the incapacity to excise the PAI affects the expression of some of genes involved in the mechanisms by which *Salmonella* can either cross or survive intracellularly in the intestinal epithelium. As a matter of fact, we observe that impairment of excision affects the transcription of genes of SPI-1 that regulate the formation of the SCV in epithelial cells (Fig 8).

In mLN, we found a high level of ROD21 excision at early stages of the infective cycle. This result suggests that the environmental conditions found in the mLN at initial stages of infection promotes ROD21 excision. Also, previous studies showed that *S*. Enteritidis increases the excision of ROD21 when growing in conditions that mimic the phagosome environment, such as low pH, high temperatures and high amounts of reactive oxygen species [28]. Considering these data, it is possible to propose that only bacteria that have underwent ROD21 excision are able to survive intracellularly in the ileum and colon and reach/colonize, in an excised state, the mLN.

A study that used a library of 54,000 transposon mutants of *S*. Enteritidis identified genes in ROD21 that are needed for the colonization of liver and spleen of BALB/c mice, including *SEN1975*, *SEN1976*, *SEN1980*, *SEN1982*, *SEN1993* and *SEN1994* [35]. Importantly, we have previously demonstrated that impairment of ROD21 excision alters the expression of some of these genes *in vitro* [33]. We showed here that all the ROD21 mutant strains evaluated have a severe defect to colonize deep organs and survive intracellularly on liver and spleen, suggesting that the excision could affect the expression of ROD21 genes and the effect of these gene products on virulence. Moreover, this process probably does not affect only the expression of genes related to the excisable island, but could also affect the expression of genes related to other pathogenicity islands. In this study, we included the evaluation of 9 genes located in ROD21 and 4 genes located in other SPIs. The data obtained *in vitro* suggest that ROD21 excision affects the expression of genes within the island, related with an increase in the transcription when the DRS and integrase-coding gene (*SEN1970*) were deleted. Similar results were obtained in the strain lacking *SEN1970* and a RDF coding gene (*SEN1998*). Unfortunately, we were not able to measure the transcription of ROD21 genes *in vivo* at 48 or 144hpi on mLN and liver, possibly because the mRNA amount of these genes was very low as compared to other genes, and therefore another more sensitive assay should be used, such as RNA-seq. However, we found that all ROD21 mutant strains of *S*. Enteritidis evaluated (specially strains unable to excise ROD21) showed an increased expression of the *invA* gene. Interestingly, the levels of *invA* transcription return to normal in the ROD21 complemented strain. We also showed that infection with *S*. Enteritidis-Δ*int*Δ*rdf* increase the expression of *invA* gene at 48 hpi and 144 hpi on mLN and liver. These results suggest that ROD21 excision could be associated with the regulation of *invA* gene in SPI-1. *invA* is essential for the assembling of T3SS-1, involved in the induction of an inflammatory environment in the gut, luminal colonization and entry of *Salmonella* into epithelial cells, as well as passage across the intestinal barrier [53, 54]. The fact that *invA* gene was overexpressed on all ROD21 strains *in vitro*, but that only the strains unable to excise ROD21 showed a defect in the intracellular survival on the intestine, blood and mLN, suggest that the excision is a dynamic process involved in the regulation of *Salmonella* gene expression *in vivo* (Fig 8).

Importantly, the differences in SPI-1 gene transcription could be related to the structure of the bacterial chromosome when the excisable ROD21 is present in the chromosome. Previous studies have described the relevance of structural organization of the chromosome to maintain the adequate expression of genes inside genomic islands [55, 56]. Some reports show that the expression of genes in SPI-1 are related to changes in the level of DNA supercoiling [57, 58]. Recently, it was shown that the supercoiling the excised pathogenicity island PPHGI.1 in *Pseudomonas syringae* pv. *Phaseolicola* plays and important role in the downregulation of an effector gene [58, 59]. Therefore, it is possible that ROD21 excision can affect chromosomal organization and structure that in turn modulates the transcription of genes within the island and other areas of the chromosome, playing a role in the regulation of the genes located in other SPIs and being an interesting aspect to study in the future. Although SPI-1 and SPI-2 are the most important PAIs associated to *Salmonella* pathogenicity, our results contribute to a growing amount of data suggesting that other genomic islands can regulate the progression of infection and even the expression of genes in SPI-1 and SPI-2.

Noteworthy, in this study we have characterized for the first time the kinetics of intragastric *S*. Enteritidis infection in C57BL/6 mice. We described that bacteria passes very quickly (within the first hour) from the stomach to the colon. Then, the major load of bacteria was observed on caecum and colon, which decreases in time and reaches its lowest level at 144 hpi. This observation suggests that intestinal colonization by *S*. Enteritidis at early stages of the infection is a transitory and self-limited event, not related to bacteria replication or development of disease, because weight loss or clinical score was not observed at early times of infection. However, at 192 hpi it is possible to detect a high number of bacteria in all the tissues evaluated in the gastrointestinal tract, which is explained by the capacity of *Salmonella* to cause re-infection of these tissues by excretion from the gallbladder. In systemic tissues, bacteria can be detected as early as 3 hpi, but sustained systemic colonization occurs between 24 to 96 hpi. Colonization of spleen and liver is modest until 144 hpi, in which a peak of bacterial burden is observed, leading to intestinal re-colonization and development of the lethal disease. These results might be important in the design of experiments that evaluate features of *S*. Enteritidis in specific tissues during the infective cycle. Moreover, this and our previous studies have shown that the transfer of ROD21 could affect the virulence of *S*. Typhimurium *in vivo* [28], suggesting that acquisition of genes contained in this pathogenicity island can increase the virulence of other *Salmonella* serovars.

In summary, this study supports the notion that PAI excision plays a role in the virulence of *S*. Enteritidis, specifically in the ability of this microorganism to cross the intestinal epithelial barrier and survive intracellularly. This observation is relevant for understanding how HGT elements can influence the regulation of host genes and contribute to the fitness of pathogenic bacteria.

## Materials and methods

### Bacterial strains and culture conditions

*S*. Enteritidis PT4 NCTC13349 (*S*. Enteritidis) and *S*. Typhimurium ATCC 14028s reference strains were originally obtained from National Collection of Type Cultures (NCTC) and kindly provided by Dr. Carlos Santiviago (Universidad de Chile, Santiago, Chile) and Dr. Guido Mora (Universidad Andrés Bello). *Salmonella* stocks were stored at -80°C in Cryobank System. To perform the infection assays, one bead from Cryobank tube was grown with agitation at 37°C in Luria Bertani (LB) overnight and then subculture was performed until OD_600nm_ equal to 0.6 was reached. Then, bacterial doses were resuspended in sterile phosphate-buffered saline (PBS).

A double mutant strain of *S*. Enteritidis (*S*. Enteritidis-Δ*int*Δ*rdf*), which lacks the genes coding for ROD21 integrase (SEN1970) and recombination directionality factor (SEN1998), was generated using the Lambda Red-mediated recombination system, as described by Datsenko and Wanner [60]. Briefly, using pKD4 and pKD3 plasmids as a template, kanamycin (*aph*) and chloramphenicol (*cat*) resistance genes were amplified by PCR using the primers SEN 1970-(H1+P1) / SEN1970-(H2+P2) for *aph* and SEN1998-(H1+P1) / SEN1998-(H2+P2) for *cat*. Electrocompetent *S*. Enteritidis harboring the thermosensitive plasmid pKD46 were electrotransformed with PCR products containing *aph* or *cat* genes. Deletion of SEN1970 and SEN1998 and antibiotic resistance lost was verified by PCR. To perform an *in vivo* competence assay, *S*. Enteritidis-WT and *S*. Enteritidis-Δ*int*Δ*rdf* strains resistant to either kanamycin or chloramphenicol were generated by the insertion of the *aph* or *cat* genes in the intergenic region of *put*A (SEN0986A) and *put*P (SEN0987), using the allele exchange technique described by Datsenko and Wanner. Other strains used in this study for *in vivo* and *in vitro* assays were previously generated in our laboratory: *S*. Enteritidis-ΔDRSΔ*int*, *S*. Enteritidis-ΔROD21 *putAP*::*cat* (*S*. Enteritidis-ΔROD21), complemented strain *S*. Enteritidis ΔROD21 *putAP*::*cat*:ROD21::*aph (S*. Enteritidis-ΔROD21::ROD21) and *S*. Typhimurium *putAP*::*cat*:ROD21::*aph* (*S*. Typhimurium::ROD21) [28, 33]. All primers used on this study are listed on Table 1.

**Table 1:** List of primers and probes used on this study to determine gene expression.

Primers and probes to test ROD21 genes				
Gene	Primer exp Fw (Sequence 5´ to 3´)	Primer exp Rv (Sequence 5´ to 3´)	Probe	Hypothetical role
SEN1970	GCC ATG CGG ATT AAG CAA TAC	TTT TGC TGG ACG GCA TGA C	TCA CCG CGA TCC TA^30^	Integrase. P4-like integrase.
SEN1975	GAA TTA AAT GGT TTG ACT GCT AGA GAG A	TCG TGC CAG ATA GGC AGT ATT ACT	AGC GGT GAA AAC C^30^	TlpA. Cytoplasmic protein with TIR domain (*Salmonella sp*.) Identities: 100%.
SEN1976	GCC GAT CCC GGA CAA GA	AGT ATC GGG CTA TAA GTG TGT AAT ACC A	CAT CCC CTT GTA TCG CT^30^	Pseudogene (putative type IV prepilin protein, *S*. Gallinarum. Identities: 99%).
SEN1979	GGC TAC TTC TGC CCC AAG AA	GTC AAT GGA GAA ATC AGA ACA AAA GA	ATC TTA AGC CTT GTT TCA AC^30^	Conserved hypothetical protein (S. Enteritidis and *S*. Dublin). Identities: 100%. (Conjugal transfer protein TraD domain).
SEN1980	TGG TCA TAT TAT ATT GGC ACC TGT TT	CAC AAT GCT GAG GAG CTT TCA A	CGC ACC TGC TCA TAT^30^	MobA/MobL family protein (*S*. Dublin) and possible conjugal transfer protein (*S*. Enteritidis). Identities: 100%. (MobA/MobL family).
SEN1982	CCA TCA GGC CCT GCT CAA	TGG CAC AGG GAC GAA TTC A	AAA ACC GTC GAG GAT C^30^	Lipoprotein (*S*. Enteritidis). Identities: 100%.
SEN1993	GCA GAA CTC CTT GCT GCT GTT	GAG CGC GGG TCG ATT TG	CTC ATC CCA ACC TAA AA^30^	DNA-binding protein (histone-like protein hlp-II) (*S*. Gallinarum and *S*. Enteritidis). Identities: 100% (Domain: global DNA-binding transcriptional dual regulator H-NS; Provisional).
SEN1994	AGT GCC TTT TGA GCC ATT GAA	TGC AAT TTA TAT CGG AGC ACC AT	ATG CCT TGT AAT TCG TG^30^	Membrane protein (S. Gallinarum and *S*. Enteritidis). Identities: 100%.
SEN1998	GAA AAC GCC CGG CCT TAA	CGC ACC GGA TTT GGT AAA A	CAG TTC GTA AAT CCA C^30^	Phage regulatory protein (*Salmonella* sp.). Identities: 100%. (Domain: Prophage CP4-57 regulatory protein (AlpA)/Predicted transcriptional regulator (Blum et al.)).

Primers and probes to test SPI-1 and SPI-2 genes			
Gene / sequence	Primer exp Fw (Sequence 5´ to 3´)	Primer exp Rv (Sequence 5´ to 3´)	Probe
*rpo*D	GTT GAC CCG GGA AGG CGA AA	CAG AAC CGA CGT GAG TTG CG	CGA CAT CGC TAA ACG [23]
*invA*	CGG TGA CAA TAG AGA AGA CAA CAA A	TTC GGG CAA TTC GTT ATT GG	CCG CCA GGC TAT C ^(This work)^
*sipB*	TGG GCA AAA ATA CGG AAG AAA	TTC CTG CAA GGC GTT GAA A	TGC AAA ACG ATC TTG CGC ^(This work)^
*spiC*	TCT TAA ATG ACC TGG AAA GCT TAC C	GGG TGT GCT GCA AGC AGT AG	AAG CCT TGT CTT GCC TAT ^(This work)^
*spiA*	AAG TAT GCC ACT GCG ATG GAT	TGG AAC CAC GAC GGA CTG AT	CCC AAT ACC AAT ATC GC ^(This work)^
*attB*	GTT ACT ATG CGC CCC GTT CAC AC	CCG ATT AAG CCC CAA AAA CTA TG	TTC GAG TCC AGT CAG AGG A [23]

### Ethics statement

All animal work was reviewed and approved by the Institution Scientific-Ethical Committees for Animal and Environmental Care and Research Biosafety, from Pontificia Universidad Católica de Chile (Protocol number 160125001), verifying that it complies the basic principles indicated in the Chilean Law 20,380 on Animal Protection (2009), the Terrestrial Animal Health Code of the World Organization for Animal Health (OIE, 24th Edition, 2015), the European Directive 2010/63 / EU and the Guide for the Care and Use of Experimental Animals (NRC, 8th Edition, 2011), documents attached to this institution. The protocol also complies with the 3Rs principle: Replace, Reduce and Refine.

### Mouse strains and infections

C57BL/6 wild type male mice (WT) from six to eight weeks old, originally obtained from Jackson Laboratories (Bar Harbor, ME), were bred and maintained in a pathogen-free animal facility at the Facultad de Ciencias Biológicas, Pontificia Universidad Católica de Chile. For *in vivo* assay to evaluate the virulence and the excision of *S*. Enteritidis WT and *S*. Enteritidis-Δ*int*Δ*rdf* across the infective cycle, sample size was calculated with the software Power and Sample Size Calculations, Version 3.0.43 using a linear regression model for experimental studies (according to the ethics committee recommendations) obtaining a sample size of 2 mice per time/strain (virulence) and 4 mice per time/strain (excision). To the other *in vivo* assays, we used the software http://stat.ubc.ca/~rollin/stats/ssize/, and assigning as a value of the mean for the null hypothesis = 1 and for the alternative hypothesis = 3 and obtain an effect size d = 3.91, considering α = 0.05 and β = 0.2, obtaining a sample size of 3 mice per group. Two independent experiments were performed, using in total 6 mice per group. Mice were lightly anesthetized with isoflurane and infected by intragastric (i.g.) route with 1x10^6^ colony forming units (CFU) of *S*. Enteritidis strains or 1x10^5^ CFU of *S*. Typhimurium strains, in 200μL of PBS. Intraperitoneal infection was performed with 1x10^5^ CFU of *S*. Enteritidis in 100μL of PBS. Administration of PBS was performed on control mice when required. Mice included in the survival studies were monitored daily for weight, illness, and signs of obvious discomfort, distress or pain. Mice that exhibited a weight loss of greater than 20% of their starting weight were euthanized via isoflurane, followed by cervical dislocation.

### Tracing ROD21 excision across infective cycle

To evaluate the ROD21 excision throughout the infective cycle of *S*. Enteritidis, 20 mice were infected with *S*. Enteritidis WT or *S*. Enteritidis-Δ*int*Δ*rdf*. 10 mice were used as control. Two infected mice per group and one control uninfected were used to determine bacterial loads and genomic DNA (gDNA) from gastric content, small intestine, caecum, colon, feces, mesenteric lymph nodes (mLN), blood, spleen, liver and gallbladder at 1, 3, 6, 24, 48, 96, 144 and 192 hours post-infection (hpi). To obtain more accurate data of the transition phase between digestive and systemic infection, an additional group of mice was included at 48 hpi.

### Bacterial load quantification

To quantify bacterial loads, infected mice were euthanized, and the organs were weighted, disrupted in PBS, serially diluted and then seeded in MacConkey (MAC) agar (Difco MacConkey, BD, USA, to digestive organs) or LB (Difco LB Agar Miller, BD, USA, to sterile organs and inoculums), using microdrop technique. Plates were incubated overnight at 37°C for 18 hours (h) and CFU were counted and calculated according to the initial weight (mg) or/and volume (ml) of the organ. Agar plates were supplemented with kanamycin (50μg/ml), chloramphenicol (10μg/ml) or ampicillin (100μg/ml) to select resistant bacteria.

### Extraction of nucleic acids and cDNA synthesis

Genomic DNA (gDNA) used in this study was prepared using the phenol-chloroform method with modifications. Homogenized organs were mixed and incubated for 45 min with lysis buffer (Ethanol 19%: Phenol 1%: SDS 0.1%, Merck Millipore) on ice and centrifuged at 8,500 x g for 10 min. The pellet was then resuspended in 570μL of TE buffer (10mM Tris-Cl (pH 8.0) 1mM EDTA (pH 8.0)), 5μL of RNAse A (Life Technologies, Invitrogen, 5mg/mL), 10μL of proteinase K (Sigma-Aldrich, 10mg/mL) and 30μL of SDS 10% and the mix was incubated at 37°C 1 h. Extraction was performed by adding an equal volume of phenol: chloroform: isoamylic alcohol (Winkler 50:48:2) and the mixture was vigorously shaken until the solution became milky. Next, the mixture was centrifuged at 21,000 x g for 10 min. The organic phase was removed and the extraction procedure was repeated twice. gDNA was precipitated by adding of 0.6 volumes of isopropanol (Merck EMSURE) and 0.1 volume of sodium acetate 3M to the aqueous phase and incubated for at least 1 h at -20°C. gDNA was centrifuged at 21,000 x g for 10 min, the supernatant was removed and the pellet was washed with 200μL of cold ethanol 70% (Merck). After 5 min of incubation at room temperature (RT), gDNA was pelleted at 21,000 x g 10 min at 4°C, the supernatant was removed, and the pellet was resuspended in 50μL of nuclease-free (NF) water (AMBION, Invitrogen). RNA extraction from bacteria was carried out with TRIzol as described by the manufacturer and reverse transcription was performed using iScript cDNA Synthesis Kit (Biorad) which was prepared according to the manufacturer´s instructions.

### Quantitative real-time qPCR

To quantify ROD21 excision, quantitative real-time PCR (qPCR) was performed using TaqMan probes and TaqMan Fast Advanced Master Mix, according to the manufacturer’s instructions for 20μL of reaction mixture. Probes and primers for *att*B-1-RT [22] and *inv*A-RT were designed using Primer Express Software v3.0.1 License (Table 1). For the quantification of excision, we used standard curves for *att*B and *invA* generated by serial dilutions, starting at 1x10^8^ genomic determinants (gDets) calculated using Avogadro´s number [61]. Since organic matrix can affect the probe sensitivity, a cut-off point was defined by each matrix, *invA* and *att*B by calculating the mean of the CT values for negative controls minus 2x standard deviation of the mean. Also, spike and recovery assay were performed to validate and assess the accuracy of qPCR for each organ (S1 Table). Relative Frequency of ROD21 excision (_RE_ROD21) was calculated using the ratio between *att*B gDets / *invA* gDets and absolute excision was expressed as *att*B or *invA* gDets adjusted per 1μg of total gDNA. Quantitative real-time PCRs (RT-qPCRs) were carried out from inoculums and organs, using specific primers and TaqMan MGB probes for ROD21 genes *SEN1970*, *SEN1975*, *SEN1976*, *SEN1979*, *SEN1980*, *SEN1982*, *SEN1993*, *SEN1994* and *SEN1998*, SPI-1 genes *invA* and *sipB* and SPI-2 genes *spiA* and *spiC* (Table 1). A StepOnePlus thermocycler was used, using the cycling conditions established for TaqMan Fast reagent. The expression of the target gene was normalized by the housekeeping gene *rpoD* and abundance of each target mRNA was determined by the comparative method (2^-ΔΔCt^).

### *In vivo* competence assay

To evaluate the ability of *S*. Enteritidis strain to colonize the organs of C57BL/6 mice, a competition assay was designed. Groups of mice were infected with a mixture of 1x10^6^ CFU of each antibiotic-resistant strain: *S*. Enteritidis-WT::*aph* and *S*. Enteritidis *S*. Enteritidis-Δ*int*Δ*rdf*::*cat*. The initial proportion of kanamycin and chloramphenicol resistant bacteria was determined by seeding inoculum on LB plates with antibiotic. After 24, 48 and 144 hpi, mice were euthanized and small intestine, caecum, colon, feces, mLN, gallbladder, livers and spleens were recovered and seeded to determine the number of CFU of each resistant strain recovered. Competition Index (CI) values were calculated as the mean ratio of *S*. Enteritidis-Δ*int*Δ*rdf* / *S*. Enteritidis-WT, normalized to the input ratio and converted logarithmically [33]. Two independent experiments were performed.

### Role of ROD21 excision on intracellular invasion and survival

To evaluate the role of ROD21 on the *S*. Enteritidis ability to invade and survive intracellularly into the organs of C57BL/6 mice, groups of mice were infected with several *S*. Enteritidis strains: *S*. Enteritidis-WT, *S*. Enteritidis-Δ*int*Δ*rdf*, *S*. Enteritidis-Δ*DRS*Δ*int*, *S*. Enteritidis-ΔROD21 or *S*. Enteritidis-ΔROD21::ROD21. After 48 and 144 hpi, mice were euthanized and small intestine, caecum, colon, feces, blood, mLN, gallbladder, livers and spleens were recovered and seeded to determine the number of extracellular loads. To determine the intracellular load, an *ex vivo* gentamicin protection assay was performed on suspensions of cells obtained from the small intestine, colon, blood, mLN, liver and spleen of infected mice. The cells were treated with 100 μg/ml gentamicin for 1 h at 37°C and seeded in plates to control the efficient killing of extracellular bacteria. Then, cells were centrifuged at 8,500 x g 10 min, washed with sterile PBS 2 times (to remove residual gentamicin), homogenized in 1 mL of 1% Triton and incubated 30 min at RT. Next, lysed cells were centrifuged at 8,500 x g 10 min and the pellet obtained was resuspended on sterile PBS. Bacterial loads were quantified by microdrop plating on LB agar. Two independent experiments were performed. To determine the effect of ROD21 intracellular ability of other *Salmonella* serovars, we performed the same experimental design described above, infecting mice with *S*. Typhimurium WT strain or *S*. Typhimurium::ROD21.

### Statistical analysis

Statistical analyses were performed using Prism v6 (GraphPad Software, San Diego CA). Unpaired Student’s t test with Holm-Sidak post-test for Multiple comparisons were used to assess whether the means of two normally distributed groups differed significantly. Two-way ANOVA analysis with repeated measures was used to compare multiple means on the experiments. Spearman´s correlation assay was performed to evaluate the correlation between CFUs load and gDets load on the different organs tested. Survival comparisons were performed using Mantel-cox test.

## Supporting information

S1 Fig Relation between quantification of *S*. Enteritidis by qPCR and CFU count in infected tissues. (A-D) *Salmonella* load was quantified by qPCR (*invA* detection, black circles) or by CFU plate count (empty circles) in several organs and then a Spearman´s correlation assay (E) was performed between CFU and gDets values for all evaluated organs with a 95% confidence interval.
(TIF)

S2 Fig Excision of ROD21 from *S*. Enteritidis chromosome during the infective cycle in Gastric content, feces and gallbladder. After intragastric (i.g.) infection of C57BL/6 mice, gDNA from gastric content (A), feces (B) and gallbladder (C) were purified. Quantification of total *Salmonella* gDets and absolute ROD21 excision in different portions of the gastrointestinal tract and deep organs was performed by the quantification of the *invA* (total bacteria, empty bars) or *attB* (excised bacteria, black bars) sequences, by qPCR. Number of copies for each sequence were normalized per μg of gDNA. Empty circles and dot line over each time post-infection are the relative ROD21 excision value _RE_ROD21(attB gDets/ invA gDets). The assay included 4 mice for each time. 2-way ANOVA with Tukey´s post-test α = 0.05; *p<0.05, **p<0.005, ***p<0.0005, ****p<0.0001.
(TIF)

S3 Fig ROD21 excision is required for *S*. Enteritidis to cause an efficient lethal disease in mice. *S*. Enteritidis-WT or Δ*int*Δ*rdf* were grown in liquid LB medium at 37°C, N minimal medium at 37°C or in LB medium at 43°C, and OD_600_ (A) or CFUs (B) were measured from time 0 until 300 min. No growth differences were found. The graph includes data of 3 independent experiments. Deletion of integrase (*SEN1970*) and RDF (*SEN1998*) genes from *S*. Enteritis result in a severe defect for ROD21 excision *in vitro* (C). Asterisks indicate differences in ROD21 excision between *S*. Enteritidis-WT and *S*. Enteritidis-Δ*int*Δ*rdf*. Unpair *t* test, α = 0.05, *P value* = 0.0011, n = 6. (D) Groups of 6 mice were infected i.g. with 1 x 10^6^ CFU of *S*. Enteritidis-WT (black circles) or *S*. Enteritidis-Δ*int*Δ*rdf* (empty triangles). Survival rate (D) and weight changes (E) were evaluated per 30 dpi and recorded on daily basis. Significant differences were observed between individuals infected with *S*. Enteritidis-WT or *S*. Enteritidis-Δ*int*Δ*rdf* and control group (gray diamonds, α or β respectively), or between them (δ). 2-way ANOVA with Tukey´s post-test α = 0.05, n = 6 mice/group. ***p<0.0005, ****p<0.0001. or Log-rank of Kaplan-Meier survival analysis, α = 0.05, were performed to found differences between weight or survival respectively.
(TIF)

S4 Fig Detection of intracellular *S*. Enteritidis in tissues at different times post-infection. *In vivo* competition assay for *S*. Enteritidis-Δ*int*Δ*rdf* versus *S*. Enteritidis-WT were performed using a total of 10^6^ CFUs of each strain administered at a 1:1 ratio i.g. Intracellular bacterial loads were evaluated, using an *ex-vivo* gentamicin protection assay, at 24, 48 and 144 hpi in intestinal tissues (A-B) that include small intestine (black bars) and colon (empty bars) or, deep organs (C-D) as mLN (black bars), blood (light grey bars), spleen (grey bars) and liver (empty bars). A and C graphs show the results obtained for *S*. Enteritidis *putAP*::*cat* and the B and D graphs show the results obtained for *S*. Enteritidis *putAP*::*aph*. In this assay, intracellular *S*. Enteritidis-Δ*int*Δ*rdf* was not detected in any tissue evaluated, at any time post-infection. The assay included 6 mice at all times post-infection. 2-way ANOVA with Tukey´s post-test α = 0.05.
(TIF)

S5 Fig ROD21 is required for systemic dissemination of *S*. Enteritidis in mice. C57BL/6 mice were i.g. infected with 1x10^6^ CFU of *S*. Enteritidis-WT (black circles), *S*. Enteritidis-ΔROD21 *putAP*::*cat* (grey circles), *S*. Enteritidis-ΔROD21 *putAP*::*cat*::ROD21::*aph* (black diamonds), *S*. Enteritidis-ΔDRSΔ*int (*grey diamonds) or *S*. Enteritidis-*int*Δ*rdf* (empty triangles). At 48 and 144 hpi bacterial loads were evaluated on small intestine (A), caecum (B), colon (C), feces (D), mLN (E), blood (F), spleen (G), liver (H) and gallbladder (I). Comparisons of bacterial loads between different times post-infection were analyzed by 2-way ANOVA with Tukey´s post-test α = 0.05. *p<0.05, **p<0.005, ***p<0.0005, ****p<0.0001. The assay included 6 mice at all times post infection.
(TIF)

S6 Fig Effect of ROD21 during the intraperitoneal infection. Groups of mice were infected intraperitoneally with 1 x 10^5^ CFU of *S*. Enteritidis-WT (black circles) or *S*. Enteritidis-Δ*int*Δ*rdf* (empty triangles) or *S*. Enteritidis-ΔROD21 *putAP*::*cat* (grey circles). Weight changes (A) and survival rates (B,C) were evaluated per 5 dpi and recorded on daily basis. Significant differences were observed between mice infected with *S*. Enteritidis-WT or *S*. Enteritidis-Δ*int*Δ*rdf* and control group (gray diamonds, α or β respectively), or between them (δ). 2-way ANOVA with Tukey´s post-test or Log-rank of Kaplan-Meier survival analysis, α = 0.05, were performed to found differences between weight changes or survival respectively. The assay included 6 mice at all times post infection.
(TIF)

S7 Fig Bacterial loads of *S*. Typhimurium and *S*. Typhimurium::ROD21 are equivalent in internal organs of infected mice. C57BL/6 mice were i.g. infected with 1x10^5^ CFU of *S*. Typhimurium WT (black circles) or *S*. Typhimurium::ROD21 (empty circles). At 48 and 72 hpi extracellular loads were evaluated on caecum (A), feces (B) and gallbladder (C). Comparisons of bacterial loads between different times post-infection were analyzed by 2-way ANOVA with Tukey´s post-test α = 0.05. No differences were found. The assay included 6 mice in each time post infection.
(TIF)

S8 Fig ROD21 excision affects the transcription of genes within *Salmonella* PAIs. Transcription of genes within ROD21: *SEN1970* (A), *SEN1975* (B), *SEN1980* (C), *SEN1993* (D) *and SEN1998* (E) or SPI-2: *spiA* (F) and *spiC* (G) were evaluated on RNA samples from inoculums of *S*. Enteritidis-WT, *S*. Enteritidis-ΔROD21, *S*. Enteritidis-ΔROD21::ROD21, *S*. Enteritidis-ΔDRSΔ*int* or *S*. Enteritidis-Δ*int*Δ*rdf*. 2-way ANOVA with Tukey´s post-test α = 0.05. *p<0.05, **p<0.005, ***p<0.0005, ****p<0.0001.
(TIF)

S1 Table qPCR spike and recovery of *invA* gene detection in gDNA from mice organs samples. gDNA samples from different organs, in a concentration of 50ng /μl, were assayed by adding 1μL of spike *Salmonella* gDNA stock solution, calculated to yield the intended 0, 1, 10 or 50 ng/μL spike concentration. Values reported for spiked samples reflect subtraction of the endogenous (no-spike) value. Recovery for spiked test samples were calculated by comparison to the measured recovery of spiked diluent control (PBS). Diluent for the diluent control, spike stock solutions and standard were the same. All values represent the average of three replicates.
(DOCX)
