# Supplementary figures and images for: Pathogenicity island excision during an infection by Salmonella enterica serovar Enteritidis is required for crossing the intestinal epithelial barrier in mice to cause systemic infection
Source: PLoS Pathog. 2019 Dec 4;15(12):e1008152. doi: 10.1371/journal.ppat.1008152 (PMC6968874; doi:10.1371/journal.ppat.1008152)

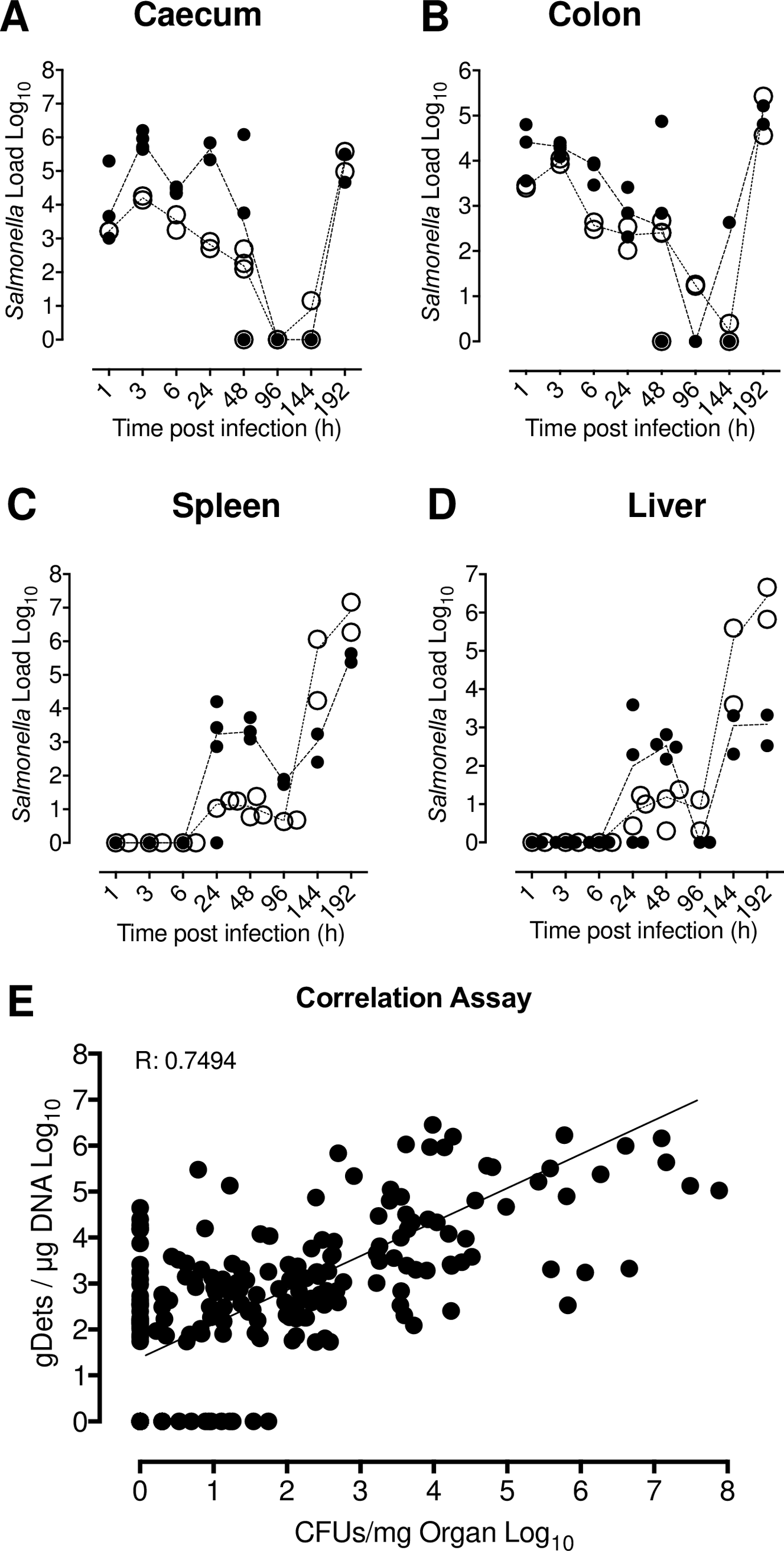

Supplement: S1 Fig — (A-D) Salmonella load was quantified by qPCR (invA detection, black circles) or by CFU plate count (empty circles) in several organs and then a Spearman´s correlation assay (E) was performed between CFU and gDets values for all evaluated organs with a 95% confidence interval. (TIF) [file ppat.1008152.s001.tif]

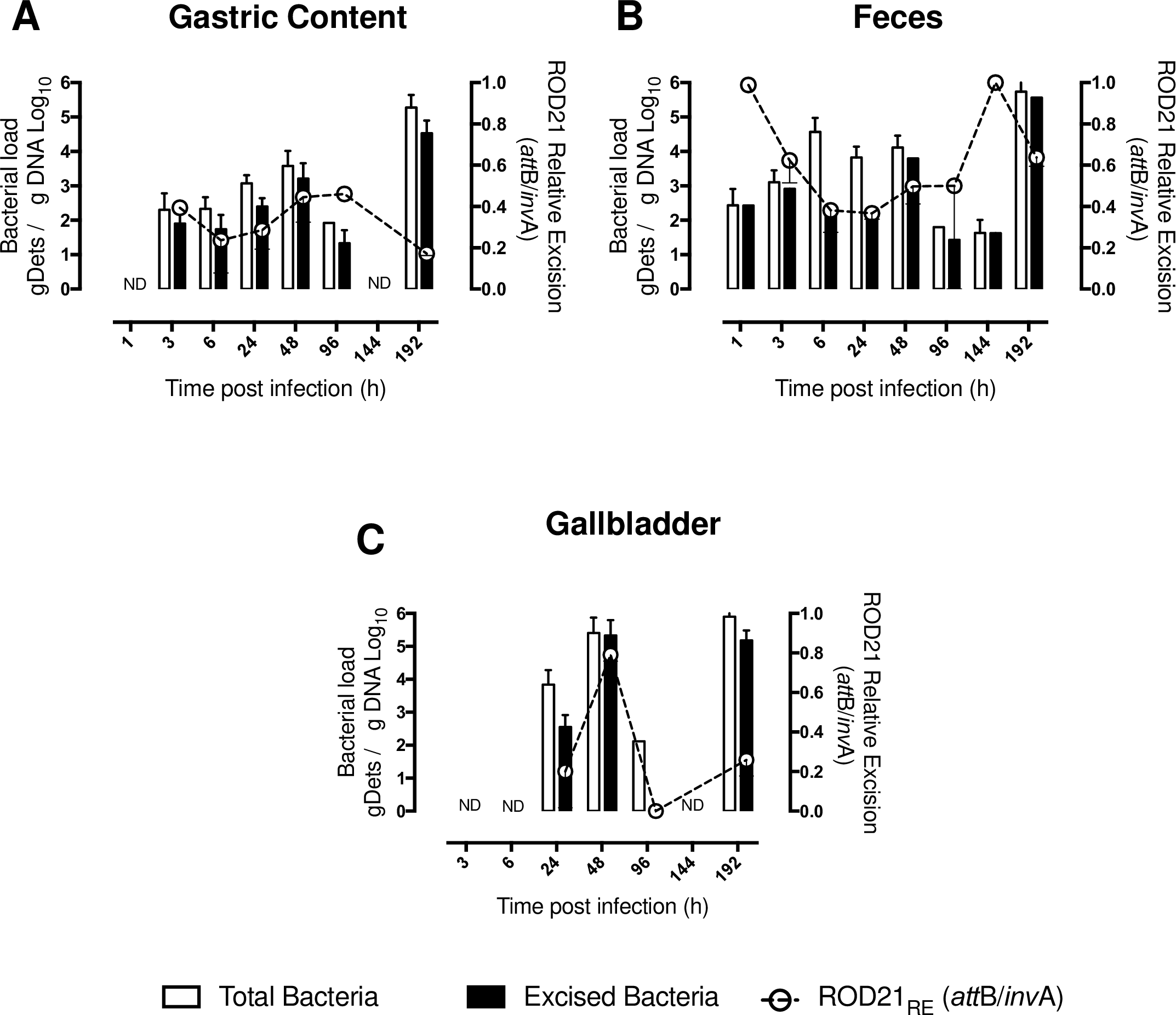

Supplement: S2 Fig — After intragastric (i.g.) infection of C57BL/6 mice, gDNA from gastric content (A), feces (B) and gallbladder (C) were purified. Quantification of total Salmonella gDets and absolute ROD21 excision in different portions of the gastrointestinal tract and deep organs was performed by the quantification of the invA (total bacteria, empty bars) or attB (excised bacteria, black bars) sequences, by qPCR. Number of copies for each sequence were normalized per μg of gDNA. Empty circles and dot line over each time post-infection are the relative ROD21 excision value REROD21(attB gDets/ invA gDets). The assay included 4 mice for each time. 2-way ANOVA with Tukey´s post-test α = 0.05; *p<0.05, **p<0.005, ***p<0.0005, ****p<0.0001. (TIF) [file ppat.1008152.s002.tif]

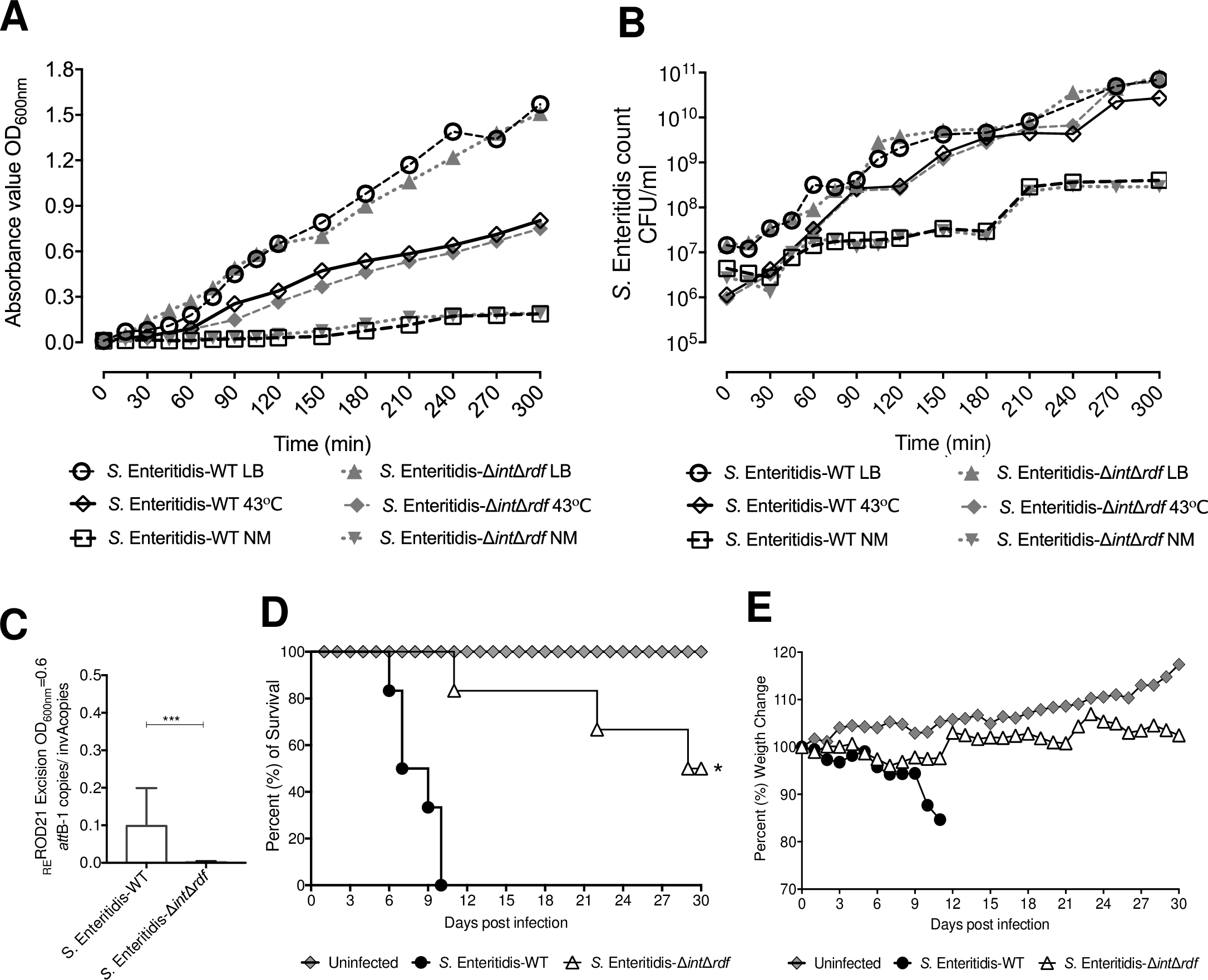

Supplement: S3 Fig — S. Enteritidis-WT or ΔintΔrdf were grown in liquid LB medium at 37°C, N minimal medium at 37°C or in LB medium at 43°C, and OD600 (A) or CFUs (B) were measured from time 0 until 300 min. No growth differences were found. The graph includes data of 3 independent experiments. Deletion of integrase (SEN1970) and RDF (SEN1998) genes from S. Enteritis result in a severe defect for ROD21 excision in vitro (C). Asterisks indicate differences in ROD21 excision between S. Enteritidis-WT and S. Enteritidis-ΔintΔrdf. Unpair t test, α = 0.05, P value = 0.0011, n = 6. (D) Groups of 6 mice were infected i.g. with 1 x 10^6^ CFU of S. Enteritidis-WT (black circles) or S. Enteritidis-ΔintΔrdf (empty triangles). Survival rate (D) and weight changes (E) were evaluated per 30 dpi and recorded on daily basis. Significant differences were observed between individuals infected with S. Enteritidis-WT or S. Enteritidis-ΔintΔrdf and control group (gray diamonds, α or β respectively), or between them (δ). 2-way ANOVA with Tukey´s post-test α = 0.05, n = 6 mice/group. ***p<0.0005, ****p<0.0001. or Log-rank of Kaplan-Meier survival analysis, α = 0.05, were performed to found differences between weight or survival respectively. (TIF) [file ppat.1008152.s003.tif]

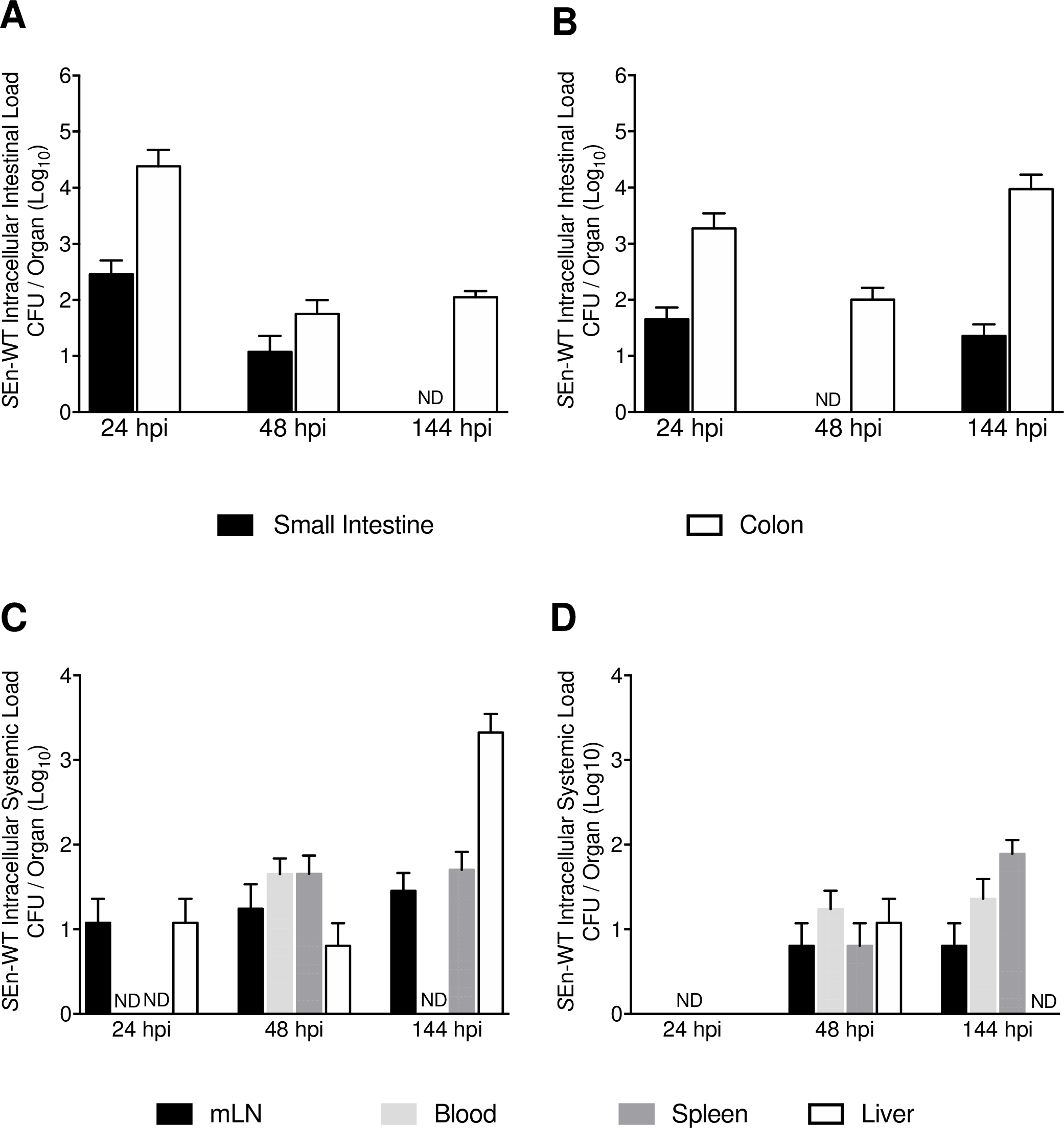

Supplement: S4 Fig — In vivo competition assay for S. Enteritidis-ΔintΔrdf versus S. Enteritidis-WT were performed using a total of 106 CFUs of each strain administered at a 1:1 ratio i.g. Intracellular bacterial loads were evaluated, using an ex-vivo gentamicin protection assay, at 24, 48 and 144 hpi in intestinal tissues (A-B) that include small intestine (black bars) and colon (empty bars) or, deep organs (C-D) as mLN (black bars), blood (light grey bars), spleen (grey bars) and liver (empty bars). A and C graphs show the results obtained for S. Enteritidis putAP::cat and the B and D graphs show the results obtained for S. Enteritidis putAP::aph. In this assay, intracellular S. Enteritidis-ΔintΔrdf was not detected in any tissue evaluated, at any time post-infection. The assay included 6 mice at all times post-infection. 2-way ANOVA with Tukey´s post-test α = 0.05. (TIF) [file ppat.1008152.s004.tif]

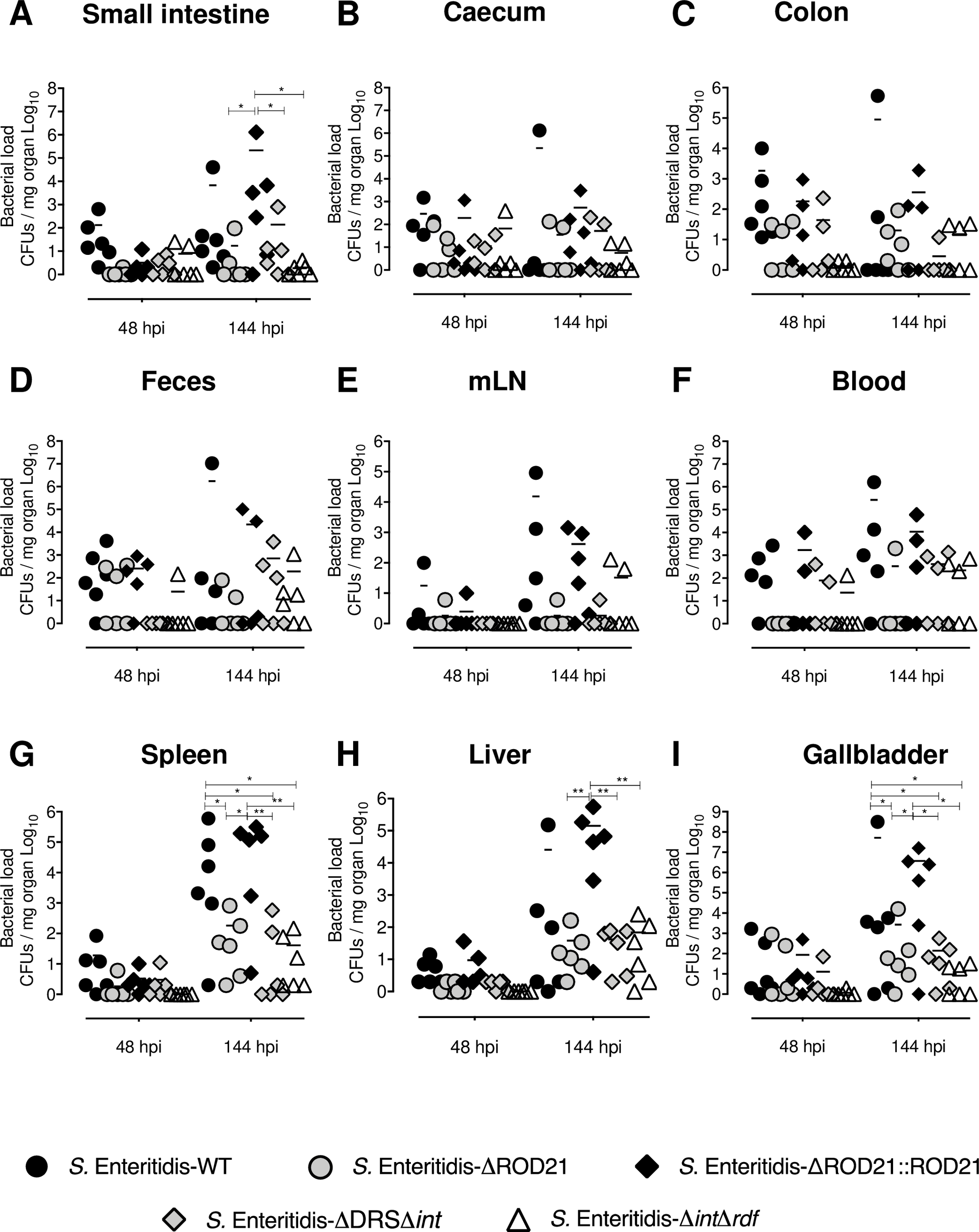

Supplement: S5 Fig — C57BL/6 mice were i.g. infected with 1x10^6^ CFU of S. Enteritidis-WT (black circles), S. Enteritidis-ΔROD21 putAP::cat (grey circles), S. Enteritidis-ΔROD21 putAP::cat::ROD21::aph (black diamonds), S. Enteritidis-ΔDRSΔint (grey diamonds) or S. Enteritidis-intΔrdf (empty triangles). At 48 and 144 hpi bacterial loads were evaluated on small intestine (A), caecum (B), colon (C), feces (D), mLN (E), blood (F), spleen (G), liver (H) and gallbladder (I). Comparisons of bacterial loads between different times post-infection were analyzed by 2-way ANOVA with Tukey´s post-test α = 0.05. *p<0.05, **p<0.005, ***p<0.0005, ****p<0.0001. The assay included 6 mice at all times post infection. (TIF) [file ppat.1008152.s005.tif]

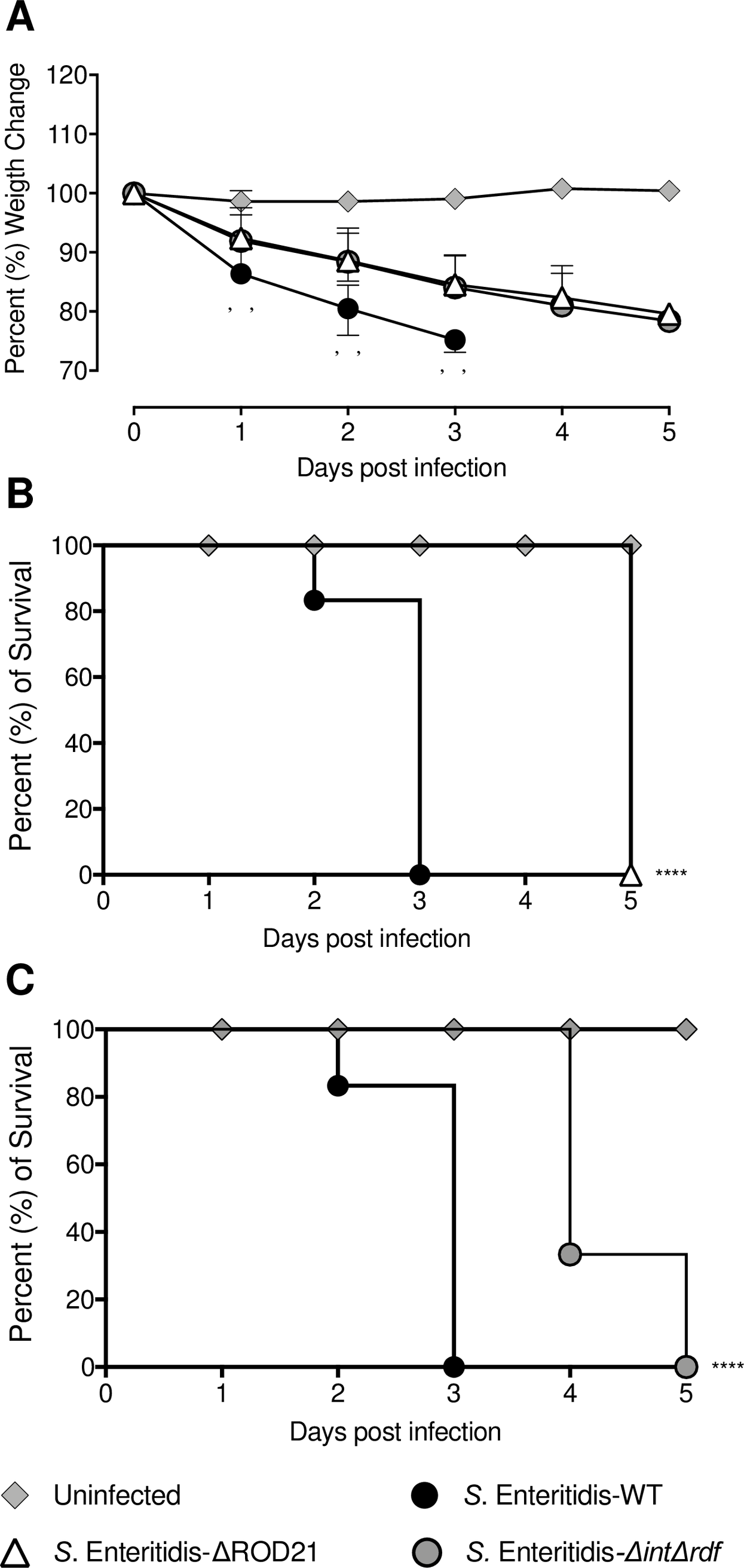

Supplement: S6 Fig — Groups of mice were infected intraperitoneally with 1 x 10^5^ CFU of S. Enteritidis-WT (black circles) or S. Enteritidis-ΔintΔrdf (empty triangles) or S. Enteritidis-ΔROD21 putAP::cat (grey circles). Weight changes (A) and survival rates (B,C) were evaluated per 5 dpi and recorded on daily basis. Significant differences were observed between mice infected with S. Enteritidis-WT or S. Enteritidis-ΔintΔrdf and control group (gray diamonds, α or β respectively), or between them (δ). 2-way ANOVA with Tukey´s post-test or Log-rank of Kaplan-Meier survival analysis, α = 0.05, were performed to found differences between weight changes or survival respectively. The assay included 6 mice at all times post infection. (TIF) [file ppat.1008152.s006.tif]

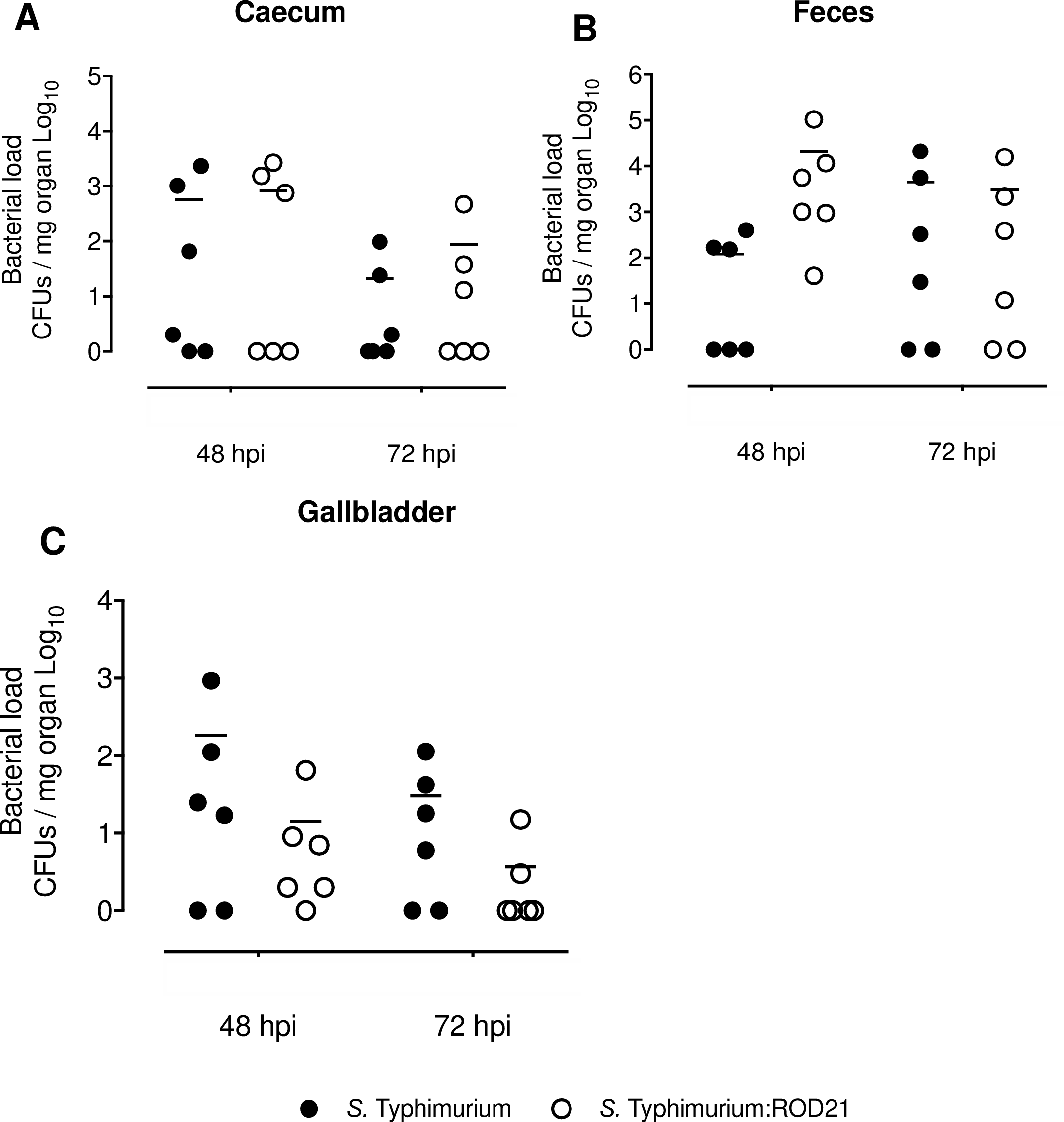

Supplement: S7 Fig — C57BL/6 mice were i.g. infected with 1x10^5^ CFU of S. Typhimurium WT (black circles) or S. Typhimurium::ROD21 (empty circles). At 48 and 72 hpi extracellular loads were evaluated on caecum (A), feces (B) and gallbladder (C). Comparisons of bacterial loads between different times post-infection were analyzed by 2-way ANOVA with Tukey´s post-test α = 0.05. No differences were found. The assay included 6 mice in each time post infection. (TIF) [file ppat.1008152.s007.tif]

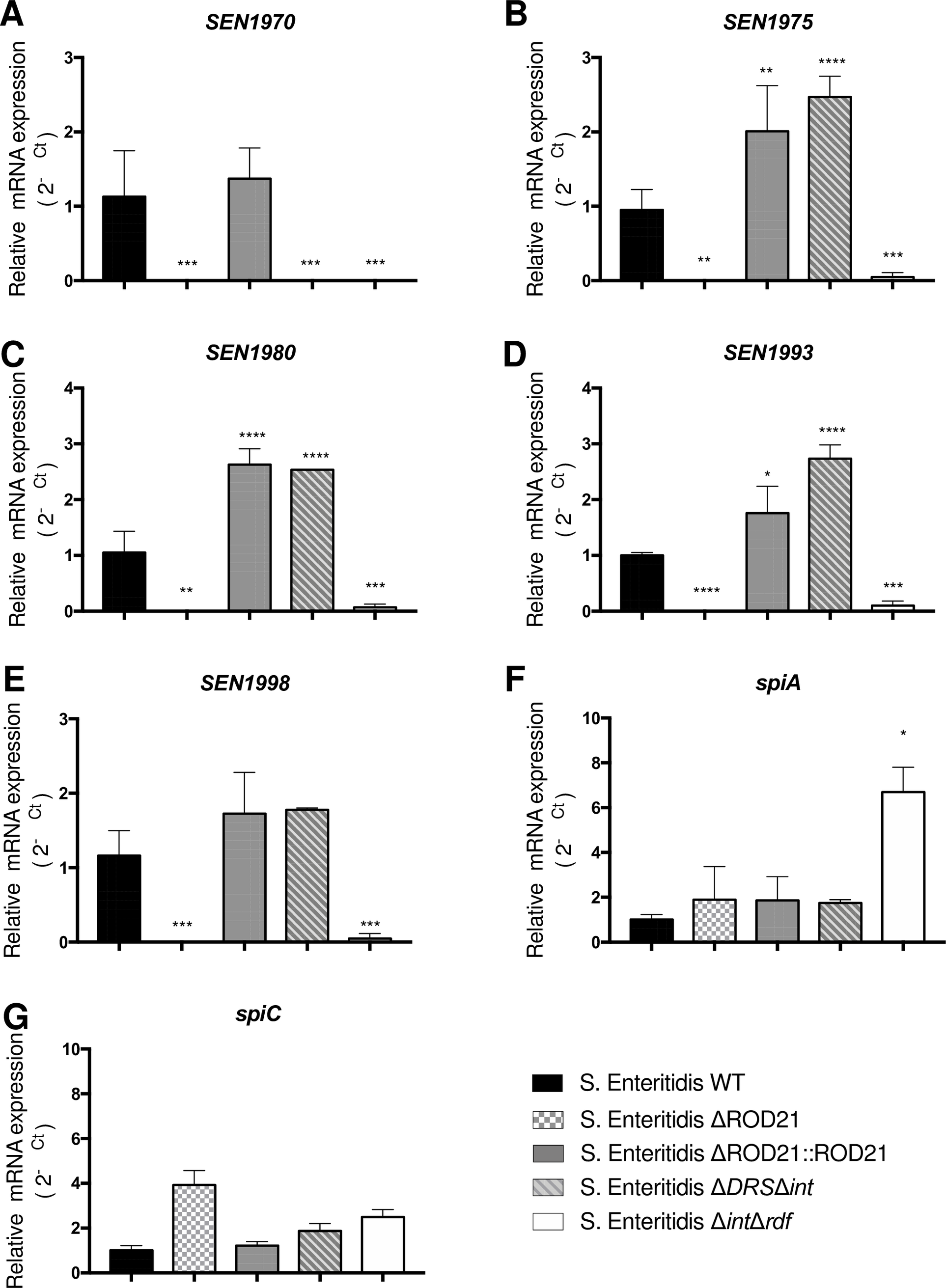

Supplement: S8 Fig — Transcription of genes within ROD21: SEN1970 (A), SEN1975 (B), SEN1980 (C), SEN1993 (D) and SEN1998 (E) or SPI-2: spiA (F) and spiC (G) were evaluated on RNA samples from inoculums of S. Enteritidis-WT, S. Enteritidis-ΔROD21, S. Enteritidis-ΔROD21::ROD21, S. Enteritidis-ΔDRSΔint or S. Enteritidis-ΔintΔrdf. 2-way ANOVA with Tukey´s post-test α = 0.05. *p<0.05, **p<0.005, ***p<0.0005, ****p<0.0001. (TIF) [file ppat.1008152.s008.tif]
