# Supplementary material for: Pathogenicity island excision during an infection by Salmonella enterica serovar Enteritidis is required for crossing the intestinal epithelial barrier in mice to cause systemic infection
Source: PLoS Pathog. 2019 Dec 4;15(12):e1008152. doi: 10.1371/journal.ppat.1008152 (PMC6968874; doi:10.1371/journal.ppat.1008152)
Supplement: S1 Table — gDNA samples from different organs, in a concentration of 50ng /μl, were assayed by adding 1μL of spike Salmonella gDNA stock solution, calculated to yield the intended 0, 1, 10 or 50 ng/μL spike concentration. Values reported for spiked samples reflect subtraction of the endogenous (no-spike) value. Recovery for spiked test samples were calculated by comparison to the measured recovery of spiked diluent control (PBS). Diluent for the diluent control, spike stock solutions and standard were the same. All values represent the average of three replicates. (DOCX) [file ppat.1008152.s009.docx]

**Table S1. qPCR spike and recovery of *invA* gene detection in gDNA from mice organs samples.**

| Spike Level | Low (1ng/μl) | | Medium (10 ng/μl) | | High (50 ng/μl) | |
| --- | --- | --- | --- | --- | --- | --- |
| Expected | 1,39 | | 10,35 | | 58,70 | |
| Samples | Observed (1ng/μl) | Recovery (%) | Observed (1ng/μl) | Recovery (%) | Observed (1ng/μl) | Recovery (%) |
| Small Intestine | 0.97 | 70.18 | 6.42 | 62.01 | 47.28 | 80.54 |
| Caecum | 1.17 | 84.24 | 10.07 | 97.25 | 47.92 | 81.63 |
| Colon | 1.09 | 78.22 | 8.06 | 77.80 | 53.91 | 91.84 |
| Feces | 1.27 | 91.67 | 9.53 | 92.01 | 49.60 | 84.49 |
| mLN | 1.30 | 93.89 | 8.92 | 86.15 | 55.01 | 93.71 |
| Blood | 0.72 | 51.81 | 7.22 | 69.75 | 47.79 | 81.41 |
| Spleen | 0.89 | 63.96 | 7.69 | 74.30 | 56.28 | 95.88 |
| Liver | 1.37 | 98.26 | 10.26 | 99.2 | 54.41 | 92.69 |
| Gallbladder | 1.20 | 86.47 | 8.42 | 81.28 | 52.06 | 88.68 |
